# Glomerular endothelial cell heterogeneity in Alport syndrome

**DOI:** 10.1038/s41598-020-67588-0

**Published:** 2020-07-10

**Authors:** Hasmik Soloyan, Matthew Thornton, Valentina Villani, Patrick Khatchadourian, Paolo Cravedi, Andrea Angeletti, Brendan Grubbs, Roger De Filippo, Laura Perin, Sargis Sedrakyan

**Affiliations:** 1 GOFARR Laboratory for Organ Regenerative Research and Cell Therapeutics in Urology, Division of Urology, The Saban Research Institute, Children’s Hospital Los Angeles, University of Southern California, 4661 Sunset Boulevard MS #35, Los Angeles, CA 90027 USA; 2 Maternal Fetal Medicine Division, University of Southern California, Los Angeles, USA; 3 Division of Nephrology, Department of Medicine, Icahn School of Medicine At Mount Sinai, New York, NY USA; 4 Nephrology Dialysis and Renal Transplantation Unit, S. Orsola University Hospital, Bologna, Italy; 5 Department of Urology, Keck School of Medicine, University of Southern California, Los Angeles, USA

**Keywords:** Chronic kidney disease, Gene expression, Glomerular diseases, Alport syndrome

## Abstract

Glomerular endothelial cells (GEC) are a crucial component of the glomerular physiology and their damage contributes to the progression of chronic kidney diseases. How GEC affect the pathology of Alport syndrome (AS) however, is unclear. We characterized GEC from wild type (WT) and col4*α*5 knockout AS mice, a hereditary disorder characterized by progressive renal failure. We used endothelial-specific Tek-tdTomato reporter mice to isolate GEC by FACS and performed transcriptome analysis on them from WT and AS mice, followed by in vitro functional assays and confocal and intravital imaging studies. Biopsies from patients with chronic kidney disease, including AS were compared with our findings in mice. We identified two subpopulations of GEC (dim^tdT^ and bright^tdT^) based on the fluorescence intensity of the Tek^tdT^ signal. In AS mice, the bright^tdT^ cell number increased and presented differential expression of endothelial markers compared to WT. RNA-seq analysis revealed differences in the immune and metabolic signaling pathways. In AS mice, dim^tdT^ and bright^tdT^ cells had different expression profiles of matrix-associated genes (*Svep1, Itgβ6*), metabolic activity (*Apom, Pgc1α)* and immune modulation (*Apelin, Icam1*) compared to WT mice. We confirmed a new pro-inflammatory role of Apelin in AS mice and in cultured human GEC. Gene modulations were identified comparable to the biopsies from patients with AS and focal segmental glomerulosclerosis, possibly indicating that the same mechanisms apply to humans. We report the presence of two GEC subpopulations that differ between AS and healthy mice or humans. This finding paves the way to a better understanding of the pathogenic role of GEC in AS progression and could lead to novel therapeutic targets.

## Introduction

The role of glomerular endothelial cells (GEC) in the pathogenesis of renal diseases is not yet well defined and better understanding of their biology could lead to discoveries of new therapeutic targets^1^. Recently, transcriptomic-profiling studies and single cell RNA-sequencing of isolated glomeruli have provided important insight into GEC heterogeneity and their potential role and adaptation to the changing microenvironment in kidney diseases^2–6^. Two recent studies based on sc-RNA sequencing of glomerular cells have identified diverse subclusters of GEC with distinct gene expression profiles. Karaiskos N. et al. identified four different subclusters of GEC based on their metabolic gene signature^2^. Dumas S.J. and colleagues on the other hand characterized GEC into five subpopulations according to differential gene expression and suggested different spatial origin within the glomerulus^6^. Other studies have shown potential adaptation of GEC to the chronically altered microenvironment during kidney disease relative to healthy. GEC from streptozotocin-induced eNOS-null diabetic mice present significant gene regulation in apoptosis, oxidative stress and proliferation pathways^5^.

In Alport syndrome (AS), a progressive renal disease associated with mutations in the COL4*α* 3, *α*4 or *α*5 chains, the potential heterogeneity of GEC and their adaptive/pathologic role is poorly understood. We have previously shown that GEC damage in AS mice precedes onset of heavy proteinuria and is characterized by endothelial fenestration changes and modulation of the VEGF signaling^7^. To study transcriptomic changes associated with GEC damage in AS, we used transgenic AS mice expressing fluorescence *td*Tomato (tdT) protein driven by the Tek promoter (Tek^tdT^ mouse). We isolated labeled-GEC from 4-month old healthy and AS mice and used pathway enrichment analysis approach to characterize their molecular signature. Both, in WT and AS mice we identified two subsets of the GEC population (dim^tdT^ and bright^tdT^) with distinct transcriptional signatures. Importantly, GEC from AS mice exhibited differential gene expression in metabolic and inflammatory response pathways versus healthy controls. Our findings from the gene regulation analysis suggest that GEC subpopulations, despite their similarities, respond differently during AS progression. We validated our findings in in vitro experiments and assessed in tissue biopsies from CKD patients, including AS.

## Results

### Characterization of GEC in WT mice and human samples

**Figure 1:**
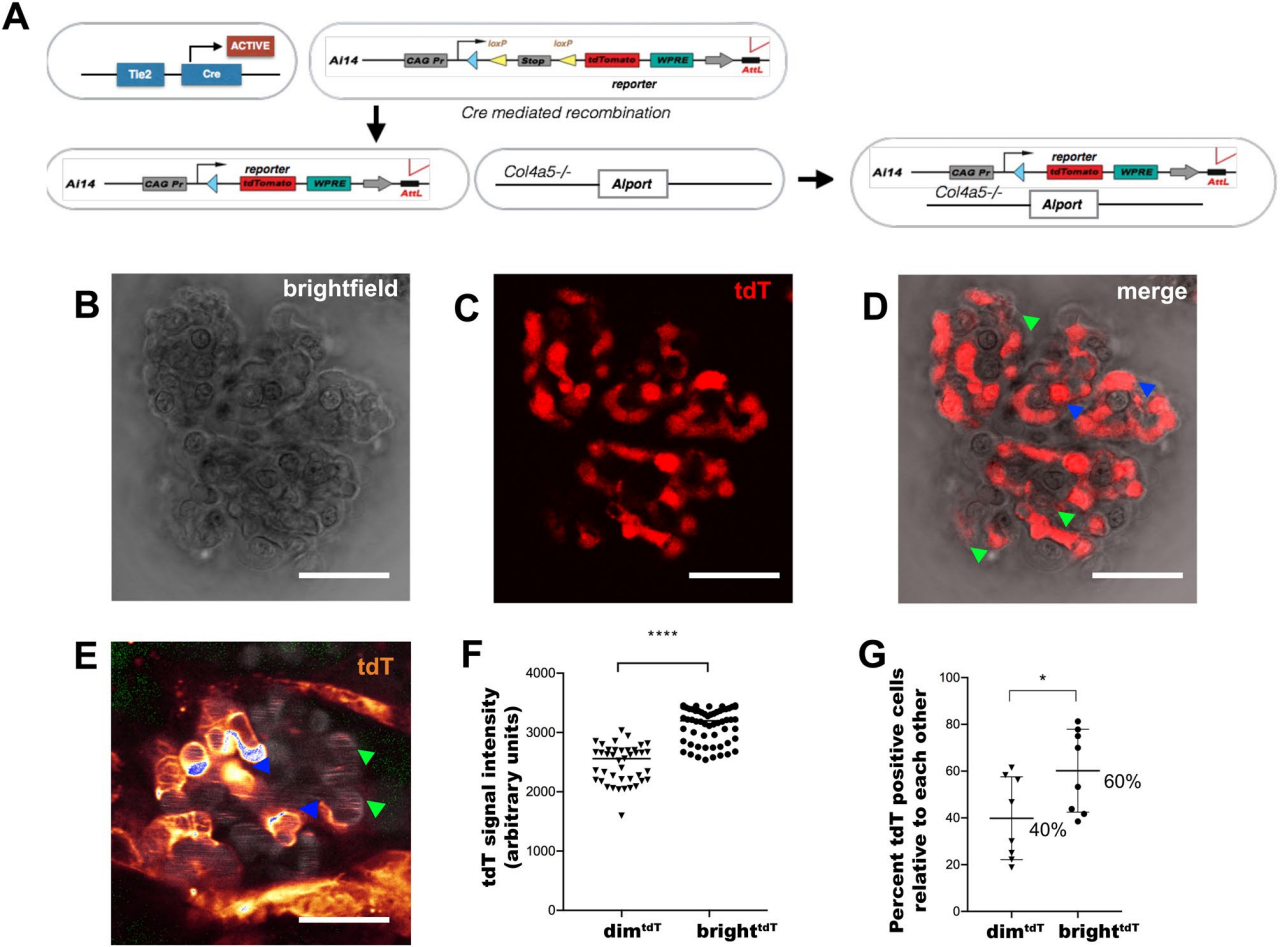
Generation and characterization of GEC specific tdT fluorescence reporter mouse. (**A)** Illustration of the breeding schemes used for generating the Tek^tdT^ transgenic reporter mouse in c57BL/6 background in AS mice. (**B–D)** Bright-field (**B**) and confocal fluorescence images (**C**) of freshly isolated tdT positive glomeruli. (An overlay image of **B** and **C** (**D**), green and blue arrowheads show the dim^tdT^ and bright^tdT^ GEC respectively) (**E, F**) A representative freeze frame image of a tdT positive glomerulus acquired using a Leica SP8 DIVE multiphoton confocal fluorescence imaging system showing the bright^tdT^ (blue arrowheads) and dim^tdT^ GEC (green arrowheads) (**E**), and dot plots showing tdT-signal intensity quantification from these images (measured as pixel density; n = 8 glomeruli) (**F**). (**G)** Dot plots showing relative percent composition of the bright^tdT^ and dim^tdT^ cell populations from (**F**). The data are presented as mean ± SD. Scale bars, 50 µm (**B**–**D**) and 25 µm (**E**). *denotes *p* value < 0.05; **** denotes *p* value < 0.0001

We used tdT reporter animals to isolate and study GEC from glomeruli (Fig. 1A). Immunofluorescence studies of glomeruli showed co-localization of Cdh5^+^ and CD31^+^ cells (endothelial markers) and co-localization of the tdT signal in Chd5^+^ cells, but not in podocytes (expressing nephrin) (Suppl. Figure 1). Since no suitable anti-Tek antibodies are available to confirm co-staining of Tek and tdT, we used Cdh5 and CD31 as a GEC specific marker and an anti-RFP (red fluorescence protein) antibody to demonstrate the endothelial specificity of the tdT signal. Brightfield and fluorescence imaging of single glomeruli revealed two types of fluorescent cells characterized by high and low intensities in the tdT signal **(**dim^tdT^ and bright^tdT^; Fig. 1B–D, Suppl. Vid.1–2). The presence of two tdT subpopulations was confirmed by two-photon intravital imaging of glomeruli in vivo (Fig. 1E) and quantified based on signal intensity (Fig. 1F, Suppl. Figure 2A) with 40% and 60% relative abundance (Fig. 1G).

**Figure 2:**
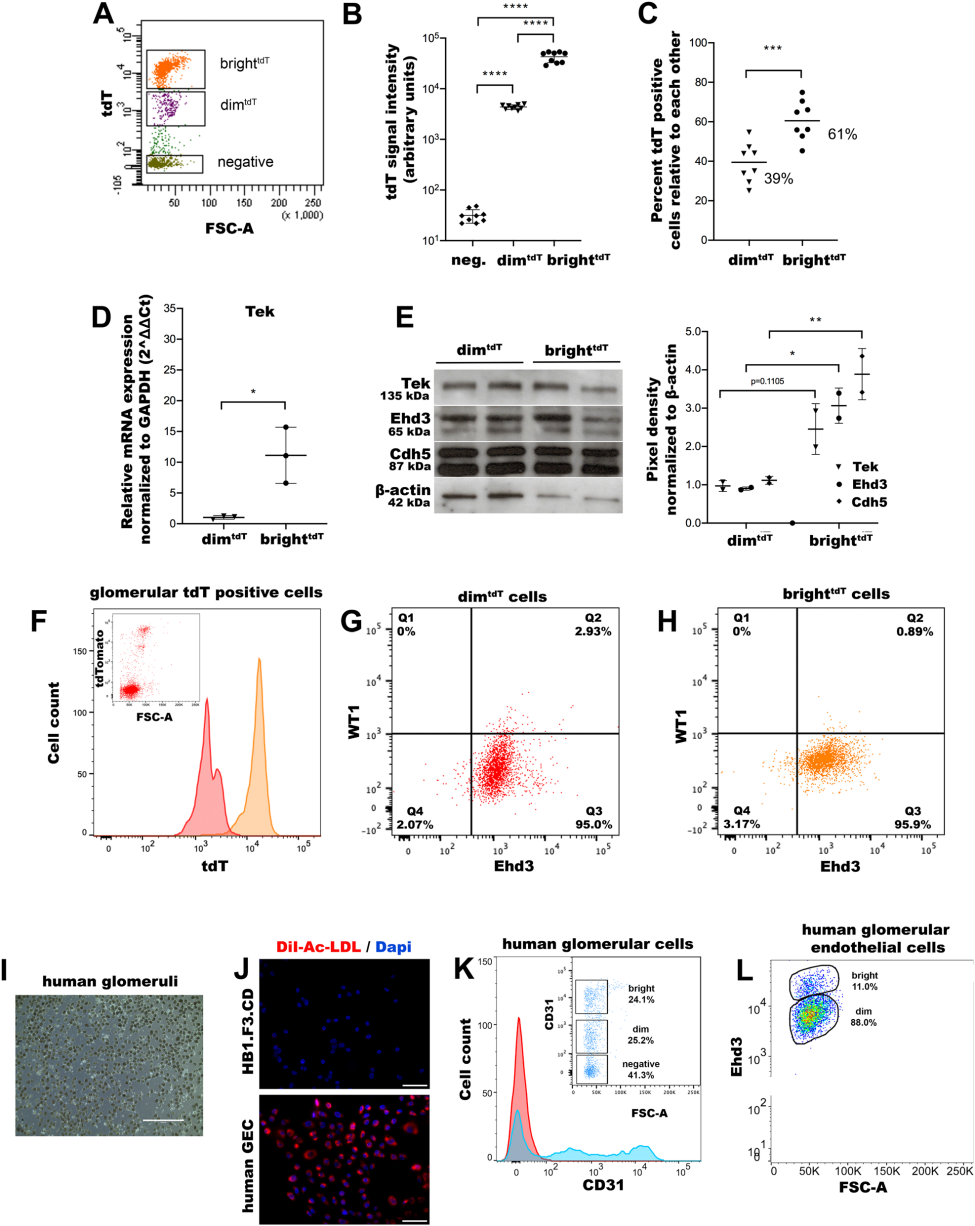
Characterization of glomerular tdTomato positive cells by flow cytometry. (**A)** Representative dot plot image of the bright^tdT^ and dim^tdT^ subpopulations as detected using the DB FACSCanto II flow cytometer. (**B, C)** Dot plots showing median intensities of the tdT signal in the negative, dim^tdT^ and bright^tdT^ cells (**B**) and relative percent compositions of dim^tdT^ (39%) and bright^tdT^ (61%) GEC subpopulations in the WT glomeruli as determined based on the total number of gated tdT positive cells (biological replicates, n = 8/group; see gating strategies in supplementary Fig. 6) (**C**). (**D)** Dot plots showing RT-qPCR analysis of Tek expression from dim^tdT^ and bright^tdT^ GEC normalized to GAPDH according to the 2∆∆^Ct^ method; biological replicates, n = 3 mice/group. € Representative immunoblots for Tek (135 kDa), Ehd3 (65 kDa) and Cdh5 (87 kDa) from dim^tdT^ and bright^tdT^ GEC normalized to β-actin (42 kDa) and densitometric analysis of the protein blots is shown in dot plots as pixel density measurements. (**F)** Mouse bright^tdT^ and dim^tdT^ GEC were flow sorted and re-analyzed for the distribution of the tdT-signal (insert image). (**G, H)** Both bright^tdT^ and dim^tdT^ subpopulations stained positive for Ehd3 (endothelial specific marker, 95% (G) and 96% (H) respectively) and were negative for WT1 (podocyte specific marker) (G-H). (**I)** A representative bright-field image of freshly purified human glomeruli. (**J)** Representative immunofluorescence images showing the characteristic uptake of Dil-Ac-LDL (red signal) in human primary GEC in contrast to human neuroblastoma cell line (HB1.F3.CD) used as a negative control. Nuclei are stained with Dapi (blue). (**K)** Human glomeruli were digested and analyzed for CD31 expression by DB FACSCAnto II flow cytometer. A representative histogram and dot plots showing the distribution of the two CD31 positive populations: bright CD31 (24.1%) and dim CD31 (25.2%). (**L)** Human GECs were further analyzed for the expression of Ehd3 protein. Representative dot plots showing the distribution of two Ehd3 positive populations: bright Ehd3 (11.0%) and dim Ehd3 (88%). The data are presented as median ± SD (B) or mean ± SD (C-E), (n = 3). Scale bars, 1,000 µm (**I**) and 100 µm (**J**). * denotes *p* value < 0.05; *** denotes *p* value < 0.001; **** denotes *p* value < 0.0001

Flow cytometric analysis of digested glomeruli also identified dim^tdT^ and bright^tdT^ GEC subpopulations, confirming histologic findings (Fig. 2A–C). The intensity difference between the dim^tdT^ and bright^tdT^ GEC was closely mirrored by the Tek expression at RNA (11.2-fold) and protein (2.5-fold) levels (Fig. 2D, E). Ehd3 and Cdh5 showed similar trend (Fig. 2E). RNA-seq results also showed 3.7-fold increase of Tek expression (Suppl. Fig. 2B). To further validate the endothelial origin of the tdT cells, we re-analyzed previously sorted dim^tdT^ and bright^tdT^ GEC by flow cytometry for tdT expression and corroborated the presence of two tdT subpopulations (Fig. 2F). Both subpopulations showed 95% or higher expression for Ehd3, while WT1 expression was virtually absent in both subpopulations (Fig. 2G, H). Consistent with our previous report, tdT signal was also absent in the mesangium^7^**,** confirming that the tdT reporter is specific to the endothelium and is devoid of any nonspecific leakage to other cell types within the glomerulus.

We next investigated human kidneys for potential GEC heterogeneity. Cells positive for CD31 were sorted by MACS and their characteristic robust uptake of Dil-Ac-LDL (specific to endothelial cells^8^) relative to a neuroblastoma cell line (HB1.F3.CD, negative control) was assessed to confirm their endothelial phenotype (Fig. 2I, J). Similar to the mouse, two subpopulations of CD31^+^ GEC were detected in freshly isolated human glomeruli (Fig. 2K). In addition, we found two subclusters of Ehd3^+^ cells in tissue-culture grown CD31^+^ human GEC (Fig. 2L), thus suggesting the presence of GEC heterogeneity also within the human kidney glomerulus.

### Characterization of GEC in AS mice

**Figure 3:**
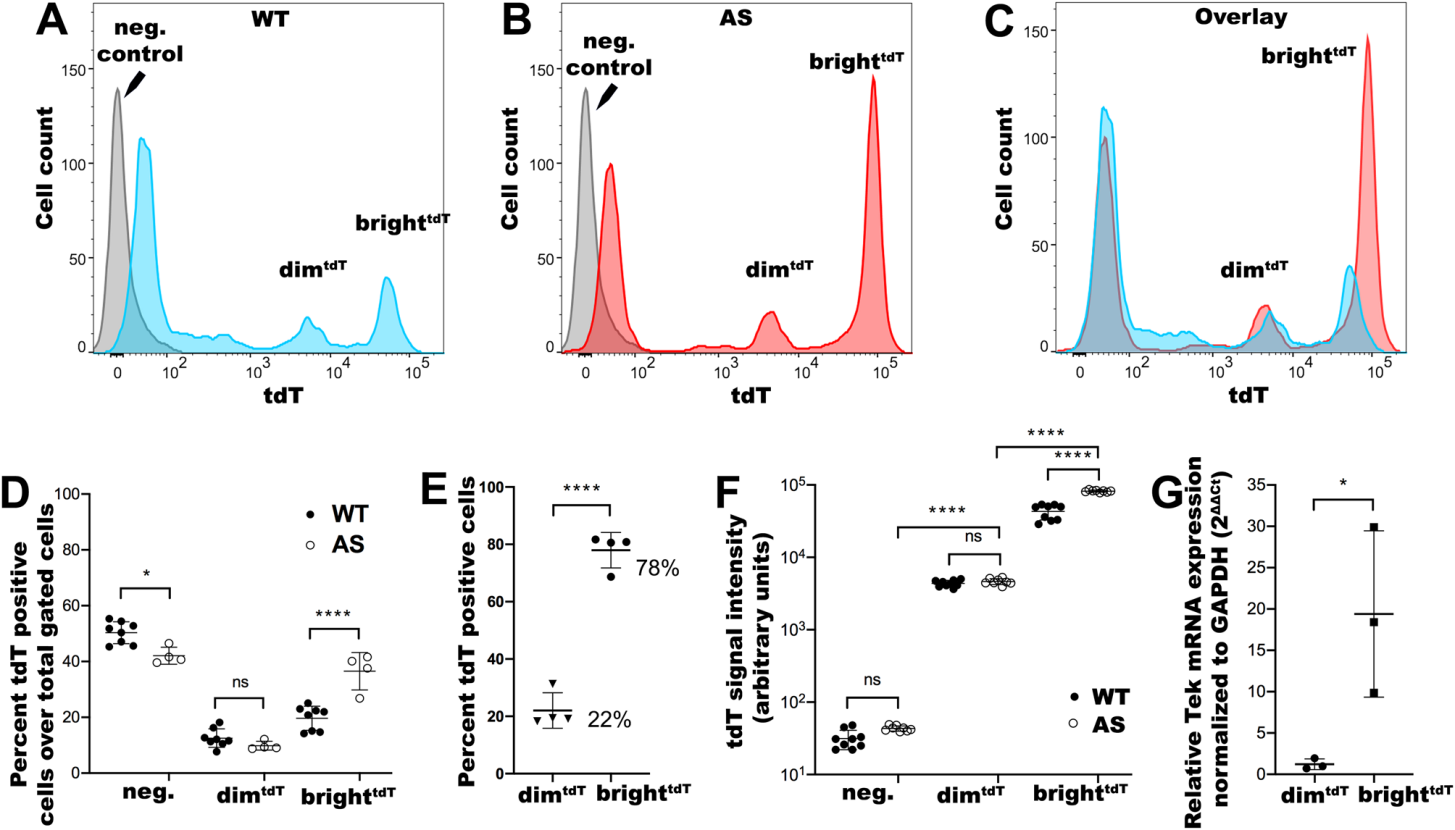
Tek-tdT expression patterns in WT and Alport glomeruli. (**A–C**) Representative histograms showing the distribution of the tdT-signal in WT (**A**) and Alport glomeruli (**B**) as measured by DB FACSCanto II and analyzed by FlowJo v10 analysis software, and their comparison (**C**). (**D**) Dot plots showing the percent distribution of the tdT-positive and -negative cell populations over total gated cells in AS glomeruli (biological replicates, n = 4 mice) in comparison to their WT counterparts (biological replicates, n = 8 mice; see gating strategies in supplementary Fig. 7). **€** (**E**). Dot plots showing relative percent compositions of dim^tdT^ (22%) and bright^tdT^ (78%) GEC subpopulations in the AS glomeruli as determined based on the total number of gated tdT positive cells (see gating strategies in supplementary Fig. 6). (**F**) Dot plots showing the tdT-signal intensities (measured in arbitrary units) for GEC subpopulations in AS (biological replicates, n = 8) and WT glomeruli (biological replicates, n = 9). (**G**) Dot plots showing RT-qPCR analysis of Tek expression from AS dim^tdT^ and bright^tdT^ GEC normalized to GAPDH according to the 2∆∆^Ct^ method; (biological replicates, n = 3 mice/group). The data are presented as mean ± SD. * denotes *p* value < 0.05; ** denotes *p* value < 0.01; *** denotes *p* value < 0.001; **** denotes *p* value < 0.0001.

In 4-month-old AS mice with CKD as documented by the presence of mild albuminuria (Suppl. Figure 2C), tdT expression identified two subpopulations of GECs similar to WT mice, except that the ratio of AS-bright^tdT^ over WT-bright^tdT^ cells were increased two-fold (Fig. 3A–D). In terms of total tdT positive cells, the relative percentage of bright^tdT^ cells were significantly higher in AS compared to WT mice (78% vs. 61%, respectively; *P* < 0.05), while the dim^tdT^ were significantly lower (22% vs. 39%; *P* < 0.05) (Fig. 2C,3E). The median intensity of bright^tdT^ GEC in AS also showed marked increase (Fig. 3F). Consistent with the protein intensity data, RNA expression of Tek in AS-bright^tdT^ GEC was significantly higher than in the dim^tdT^ and much higher than in the WT as shown by RNA-seq (Suppl. Figure 2B) and further confirmed by RT-qPCR (Fig. 3G). These changes in GEC subpopulations in AS mice might be one of the early indications of their participation to the changing milieu of the glomeruli during progression.

### Transcriptome-wide analysis of GEC in WT and AS mice

To better understand the biology of GEC subsets in AS and healthy mice, we performed genome-wide transcriptome analysis of the two tdT GEC subpopulations in three biological replicates of AS and WT sex-matched mice at 4-months of age. The following groups were compared: A) WT-bright^tdT^ over WT-dim^tdT^ GEC, B) AS-bright^tdT^ over AS-dim^tdT^ GEC, C) AS-bright^tdT^ over WT-bright^tdT^ GEC, and D) AS-dim^tdT^ over WT-dim^tdT^ GEC.

#### *Gene expression patterns in bright*^*tdT*^* over dim*^*tdT*^* in WT and AS mice*

**Figure 4:**
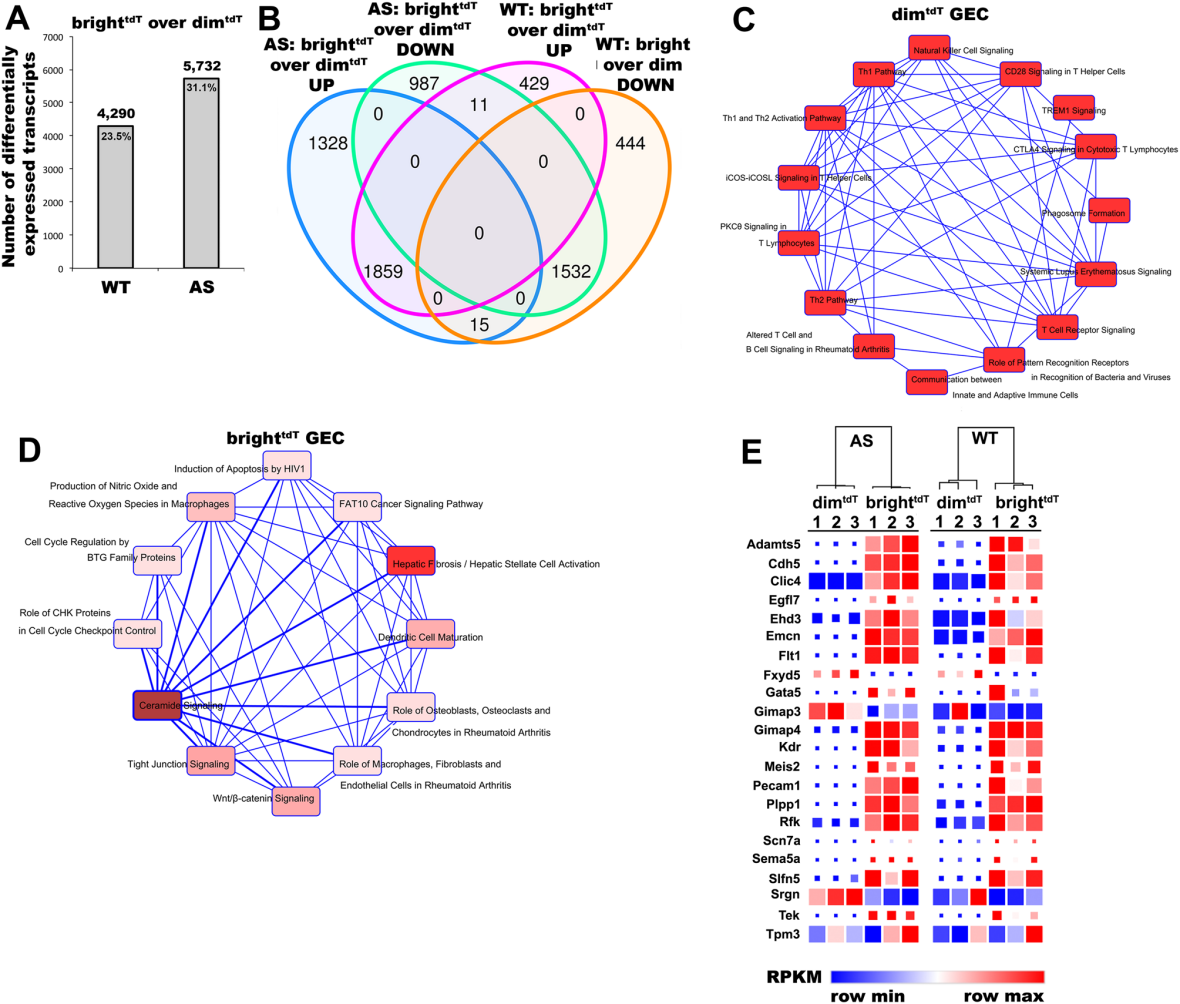
Transcriptome-wide comparison of GEC subpopulations. (**A)** Bar graphs showing the number of genes with differential expression and associated percentages in bright^tdT^ GEC compared to dim^tdT^ GEC in both WT and AS mice. (**B)** Venn diagram comparison of gene expression in bright^tdT^ cells relative to dim^tdT^ cells in WT and AS mice. Each of the four circles represents a set of genes differentially expressed between the two subpopulations; (blue and purple circles denote genes upregulated in bright^tdT^ over dim^tdT^; green and orange circles denote genes downregulated in bright^tdT^ over dim^tdT^). Numbers depicted in the intersections between circles represent the numbers of genes that are commonly up or down regulated in those groups. (**C, D)** Network graphs showing the canonical molecular pathways defining the 308 transcripts solely expressed in the dim^tdT^ cells (**C**), and the 33 transcripts solely expressed in the bright^tdT^ cells (**D**) based on the IPA software analysis (Qiagen); color scale – red color intensity indicates stronger expression. (**E**) Heatmap diagrams with color-coded representation of the RPKM values comparing the transcriptional expression of endothelial specific genes between the bright^tdT^ and dim^tdT^ GEC in WT and between bright^tdT^ and dim^tdT^ AS mice. A comparison between WT and AS is presented in Fig. 8F. The color scale represents relative gene expression levels across each row with red denoting upregulation and blue denoting downregulation. Color intensity indicates stronger regulation. *P* value < 0.05 was applied as a cut-off point for all transcriptomic data analysis (biological replicates, n = 3/group).

Differential gene expression (DGE) between GEC in group (A) was 23.5%, corresponding to a total of 4,290 genes. As predicted, group (B) had higher heterogeneity at 31.1% corresponding to a total of 5,732 genes, (Fig. 4A, B). Transcripts exclusively expressed in the dim^tdT^ (Suppl. Table 1) were significantly enriched for genes involved in activation of immune cells, including T lymphocytes (Fig. 4C). Those expressed only in the bright^tdT^ (Suppl. Table 2) were enriched in immune and metabolic pathways, especially in ceramide signaling (Fig. 4D), an important mediator of reactive oxygen and nitrogen species-triggered cell responses, like apoptosis ^9^. Genes with diverse functions that were inversely regulated in groups (A) and (B), are shown in Table 1.

**Table 1:** List of genes inversely regulated in GEC subpopulations between WT and AS mice.

WT			AS	
Genes	Bright^tdT^ over dim^tdT^	*p* value	Bright^tdT^ over dim^tdT^	*p* value
Ankrd37	1.404	0.0450	−1.64	0.0036
Arg1	3.398	0.0000	−1.715	0.0008
Nudt1	1.401	0.0300	−1.729	0.0056
Plet1	1.944	0.0090	−1.524	0.0091
Pleppr3	2.882	0.0018	−1.981	0.0166
Purg	1.392	0.0413	−1.259	0.0019
Serpinf1	2.718	0.0053	−2.035	0.0000
Stx19	2.481	0.0005	−1.869	0.0002
Ttll3	1.866	0.0220	−3.948	0.0002
Wdr95	2.691	0.0113	−1.402	0.0259
Zbtb16	1.617	0.0323	−1.397	0.0081
A4galt	−1.958	0.0323	4.402	0.0000
Aif1l	−1.119	0.0465	1.073	0.0033
Ak1	−1.251	0.0412	1.890	0.0000
Col1a2	−1.312	0.0251	1.194	0.0028
Efna4	−2.971	0.0183	3.446	0.0000
Enpep	−1.660	0.0132	1.323	0.0027
Grin3a	−2.858	0.0018	1.521	0.0113
Loxl2	−1.376	0.0374	4.382	0.0000
Oplah	−1.421	0.0269	1.671	0.0012
Polr2f	−1.673	0.0214	1.122	0.0191
Rassf10	−2.051	0.0012	1.353	0.0225
Rbpms2	−1.306	0.0278	1.778	0.0016
Rdh1	−2.638	0.0008	1.516	0.0188
Trpc6	−3.317	0.0359	5.304	0.0000
Tyro3	−2.910	0.0129	1.413	0.0106

#### *Endothelial specific gene profiles in WT-Tek*^*tdT*^

Hierarchical clustering analysis of endothelial-specific transcripts yielded two sub-clusters of cells in WT and AS mice corresponding to the dim^tdT^ and bright^tdT^ subsets, respectively and the bright^tdT^ cells showed higher relative gene expression (Fig. 4E). In contrast, the dim^tdT^ cells had stronger CD133 expression, which might suggest about their endothelial progenitor-like nature ^10^ (Suppl. Figure 2D).

#### *Gene expression patterns in bright*^*tdT*^* and dim*^*tdT*^* between AS and WT mice*

**Figure 5:**
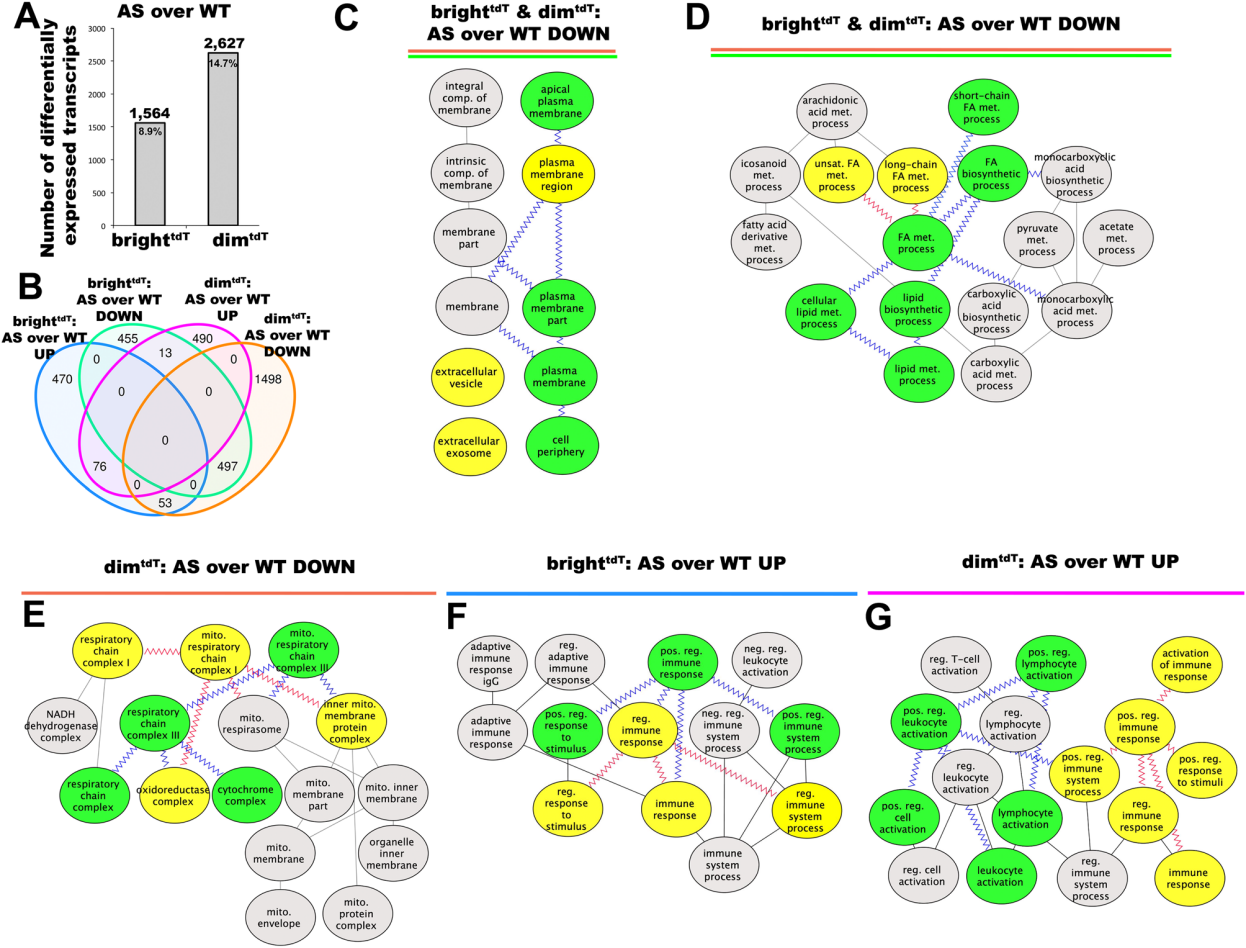
Transcriptional profiling and pathway enrichment analysis for dim^tdT^ and bright^tdT^ subpopulations in AS relative to WT. (**A**) Bar graphs showing the number of genes with differential expression and associated percentages in bright^tdT^ GEC in AS compared to bright^tdT^ GEC in WT and dim^tdT^ GEC in AS compared to dim^tdT^ GEC in WT mice. (**B**) Venn diagram comparison of gene expression, where each of the four circles represents a set of genes differentially expressed in AS GEC subpopulations relative to their WT counterparts; (blue and purple circles denote genes upregulated, while green and orange circles denote genes downregulated in bright^tdT^ and dim^tdT^ respectively in AS over WT). Numbers depicted in the intersections between circles represent the numbers of genes that are commonly up or down regulated in those groups. (**C–G**) Directed acyclic graphs of GO terms based on pathway enrichment analysis of subsets shown in Venn diagram in (**B**) showing the 497 transcripts commonly downregulated in both dim and bright GEC with significant enrichment in extracellular vesicle activity (**C**) and lipid metabolimics (**D**), the 1,498 transcripts downregulated in the dim^tdT^ GEC only with significant enrichment in mitochondrial structural and functional pathways (**E**), the 470 transcripts upregulated in the AS bright^tdT^ cells only with significant enrichment in cytoskeleton organization and cell–cell adhesion processes (**F**), the 490 transcripts upregulated in the AS dim^tdT^ cells only with significant enrichment in chemokine production and secretion pathways (**G**). *P* value < 0.05 was applied as a cut-off point for all transcriptomic data analysis (biological replicates, n = 3/group). Color-coded nodes and edges of the acyclic graphs highlight the GO terms with close associations.

The DGE between GEC in groups (C) (bright^tdT^: AS over WT) and (D) (dim^tdT^: AS over WT) were 8.9% and 14.7% corresponding to a total of 1,564 and 2,627 genes respectively (Fig. 5A). As shown in Fig. 5B, expression of 66 transcripts between the dim^tdT^ and bright^tdT^ cells were inversely regulated (Suppl. Figure 3A–B) and 53 of them were consistently enriched for biological processes involved in extracellular matrix (ECM) modeling, cell adhesion and angiogenic processes (Suppl. Figure 3C).

### Bright^tdT^ and dim^tdT^ GEC from AS have distinct immune modulatory and metabolic pathway enrichment profiles

In AS, out of the 1.75 × 10^4^ total transcripts analyzed 497 transcripts commonly downregulated in both dim^tdT^ and bright^tdT^ GEC were highly enriched in genes and signaling pathways associated with plasma membrane, extracellular vesicles and lipid metabolomics (Fig. 5C, D; Suppl. Table 3). In addition, 1,498 transcripts downregulated in the dim^tdT^ GEC were highly enriched in mitochondria associated pathways (Fig. 5E; Suppl. Table 4). 455 transcripts downregulated in the bright^tdT^ GEC showed no significant mechanistic associations. In contrast, 470 genes exclusively upregulated in the bright^tdT^ and 490 in the dim^tdT^ GEC showed consistent enrichment in GO terms strongly associated with the positive regulation of the immune responses including leukocyte and lymphocyte activation (Fig. 5F, G). In particular the bright^tdT^ GEC were more highly enriched in genes and pathways regulating cytoskeleton organization and cell–cell adhesion processes (Suppl. Table 5). In contrast the dim^tdT^ GEC were more highly enriched in genes and pathways related to chemokine production and secretion (Suppl. Table 6).

### Apelin activates inflammatory genes in GEC

**Figure 6:**
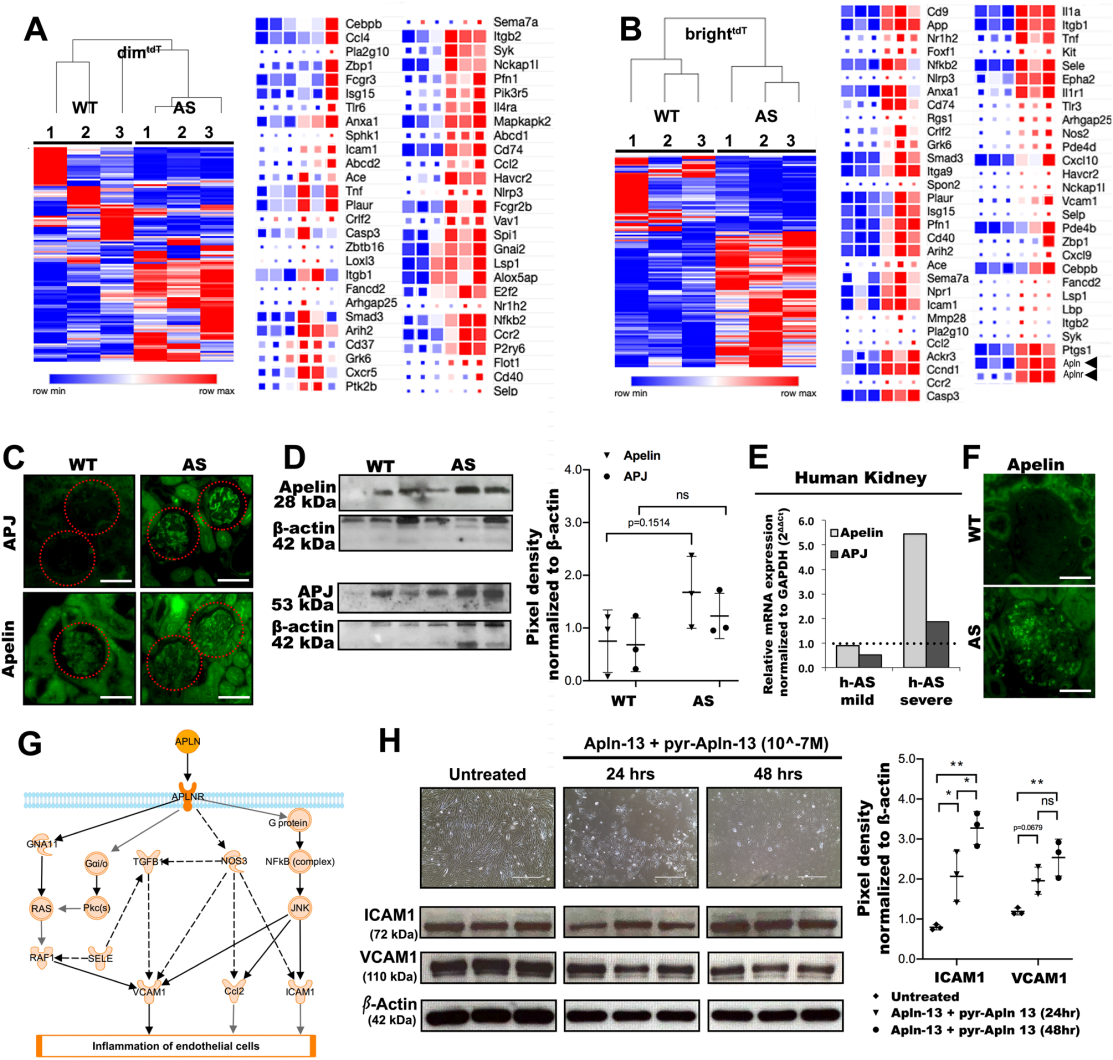
Inflammatory profile of GEC subpopulations in AS and the role of Apelin signaling. (**A, B)** Heatmap diagrams with color-coded representation of RPKM values comparing the gene expression of inflammatory markers in AS dim^tdT^ (**A**) and AS bright^tdT^ GEC (**B**) and their wild type counterparts (biological replicates, n = 3 mice/group). Genes of significance and with high differential expression are listed next to the diagram. The color scale represents relative gene expression levels across each row with red denoting upregulation and blue denoting downregulation. Color intensity indicates stronger regulation. (**C)** Representative immunofluorescence images of glomeruli from WT and AS kidney sections stained for Apelin and APJ show increased expression (green signal) in the AS. (**D)** Representative immunoblots comparing protein expression for Apelin (28 kDa) and APJ (53 kDa) in GEC between WT and AS mice normalized against β-actin (42 kDa). Densitometric analysis of the immunoblots is shown in dot plots as pixel density measurements, (biological replicates, n = 3/group). € Bar graphs showing RT-qPCR analysis and relative expression of Apelin and APJ gene expression in human kidney tissue samples from AS patients with low and high level of proteinuria (AS-mild patient (n = 1): 17 years male, proteinuria: 2.5 g/day; AS-severe patient (n = 1): 22 years male, proteinuria: 10 g/day) as compared to a healthy donor and normalized to GAPDH according to the 2^∆∆Ct^ method. (**F)** Representative immunofluorescence images of human kidney sections showing increased expression for Apelin (green signal) in the AS glomeruli compared to healthy control. (**G)** Apelin/APJ signaling cascade that leads to the activation and upregulation of pro-inflammatory genes. (**H)** Apelin-13 induces expression of inflammatory markers in human primary GEC. 2.5 × 10^5 human primary GEC were co-treated with 10^-7^ M apelin-13 and pyr-apelin-13 isoforms simultaneously for 24 and 48 h or were left untreated (control). Representative brigt-field light microscopy images show significant cell detachment and changes in cell morphology post treatment, while representative immunoblots show the time-dependent expression of ICAM1 (72 kDa) and VCAM1 (110 kDa) in the Apelin treated cells normalized against β-actin (42 kDa). Densitometric analysis of the immunoblots for VCAM1 and ICAM1 protein levels is shown in dot plots as pixel density measurements, (biological replicates, n = 3/group). Scale bars, 50 µm. The data are presented as mean ± SD. * denotes *p* value < 0.05; ** denotes *p* value < 0.01; *** denotes *p* value < 0.001.

Little is known about GEC and potential inflammation in AS. The pathway enrichment analysis suggested that immune system mechanisms are regulated in GEC (Fig. 5F, G). The AS-dim^tdT^ GEC overexpressed gene sets responsible for chemokine production and secretion (Fig. 6A). In contrast, expression of many inflammatory genes including *Icam1*, *Vcam1*, *Ccl2*, *Spon2* and *Sele* were increased in the AS-bright^tdT^ GEC (Suppl. Table 7), which correlated with marked upregulation of *Apelin* (*Apln*) and its receptor (*Aplnr*) (Fig. 6B, black arrowhead). AS kidneys stained strongly for both proteins (Fig. 6C), and slight differences was measured also by immunoblot analysis in GEC (Fig. 6D). Indeed, Apelin/APJ signaling pathway was among the top networks highly activated in bright^tdT^ GEC as determined by the Ingenuity Pathway Analysis (IPA) (Suppl. Figure 4A). Importantly, *Apln* was also upregulated in kidney biopsies from patients with advanced AS, as shown by RT-qPCR (Fig. 6E) and immunohistochemistry (Fig. 6F). Pro-inflammatory markers, such as ICAM1 and VCAM1 are found downstream of Apelin/APJ signaling cascade and activation of this pathway can potentiate their upregulation (Fig. 6G). To test if this mechanism works in GEC we used freshly isolated primary human GEC (hGEC). Stimulations with two different Apelin isoforms at 10^-7^ M (Apln-13, or *pyr*-Apln-13) independently did not affect ICAM1 and VCAM1 expression levels in cultured hGEC (Suppl. Figure 4B). However, co-stimulation with both isoforms with a combined concentration of 10^−7^ M increased their expression after 48 hours (Fig. 6H), suggesting that Apelin-13 isoforms are linked to inflammatory gene expression in GEC.

### Energy metabolism: a source of endothelial dysfunction in AS glomeruli

**Figure 7:**
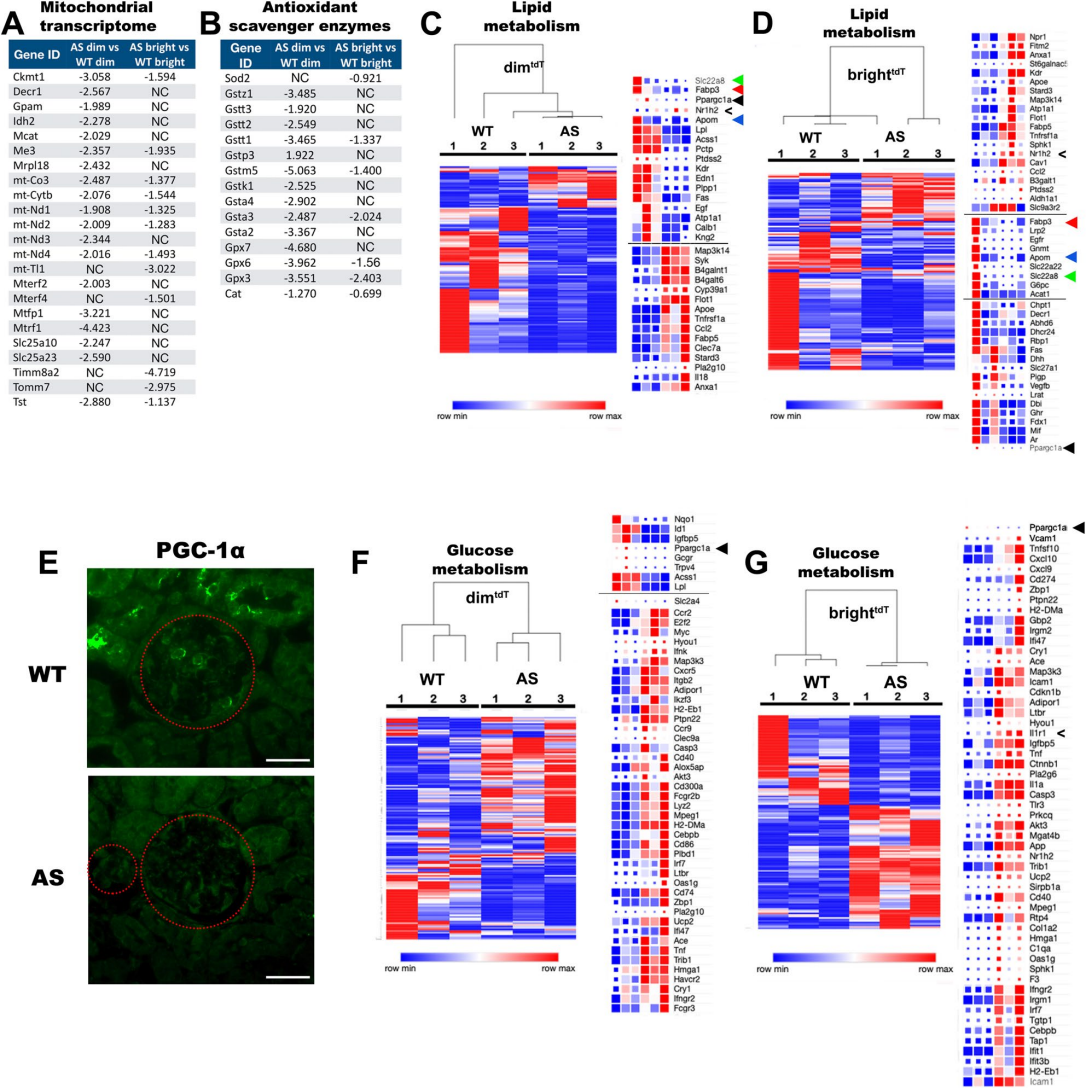
Metabolic hetergeneity of GEC in AS mice. (**A, B)** Charts showing the relative fold change expression levels of mitrochondrial genes enriched for oxidative phosphorylation (**A**) and antioxidant enzymatic scavengers (**B**) with consistent downregulation predominantly in the dim^tdT^ GEC in AS mice. (**C, D)** Heatmap diagrams with color-coded representation of RPKM values comparing the expression of glucose metabolism associated transcriptome of AS dim^tdT^ (**C**) and AS bright^tdT^ (**D**) and their wild type counterparts respectively. Genes of significance and with high differential expression are listed next to each heatmap. (**E**) Representative immunofluorescence images of mouse kidney sections stained for PGC1α in WT and AS. (**F, G)** Heatmap diagrams with color-coded representation of RPKM values comparing the expression of lipid metabolic gene activity of AS dim^tdT^ (**F**) and AS bright^tdT^ (**G**) and their wild type counterparts respectively. Genes of significance and with highly differential expression are shown next to each heatmap. Arrowheads highlight specific genes in the list. The color scale represents relative gene expression levels across each row with red denoting upregulation and blue denoting downregulation. Color intensity indicates stronger regulation. The list of genes included in the lipid and glucose metabolic panels were generated based on the Ingenuity Pathway Analysis of the total RNA-seq data. *p* value < 0.05 was applied as a cut-off point for all transcriptomic data analysis (biological replicates, n = 3/group).

Among differentially expressed transcripts between AS and WT mice, genes with well-established functional roles in mitochondrial dysfunction, glucose and lipid metabolism were most significantly enriched. Genes enriched for oxidative phosphorylation were consistently downregulated in GEC of AS mice, and more drastically in the dim^tdT^ than in the bright^tdT^ (Fig. 7A). In addition, antioxidant enzymatic scavenger gene expressions were decreased predominantly in the dim^tdT^ (Fig. 7B). *Ppargc1α* (*Pgc1α*), which co-regulates mitochondrial biogenesis and expression of several mitochondrial antioxidant enzymes and plays a key role in the protection against oxidative stress by supplying undamaged mitochondria and enhancing ROS-defenses ^11,12^ was also downregulated (Fig. 7C–D, black arrowheads, Fig. 7E). *Pgc1α* also regulates other genes related to lipid and glucose metabolism, fatty acid and glucose transport into the cells^13^. Indeed, in AS mice changes in gene expressions associated with lipid metabolism were evident in the subpopulations of GEC. Large sets of genes associated with lipid metabolism were highly downregulated in both groups (Fig. 7C, D). *Apom*, a newly discovered lipoprotein mainly expressed in liver and kidney and involved in reverse transport of cholesterol and other fatty acids from the cytoplasm to the nucleus and also shown to be associated with vascular permeability^14^ was downregulated in both subpopulations (Fig. 7C, D, blue arrowheads). Loss of *Apom* expression correlated well with upregulation of its negative regulators, *Nr1h2*, and *Il1α* (Fig. 7C, D, arrow marks)^15^. *Fabp3*, which facilitates transport of lipids to specific compartments in the cell^16^, was drastically reduced in both AS-bright^tdT^ and AS-dim^tdT^ GEC (Fig. 7C, D, red arrowheads). *Slc22a8* downregulated in AS GEC (Fig. 7C, D, green arrowhead), is a novel kidney transporter that mediates the uptake of small molecule anions^17^. Deficiency of *Slc22a8* has been associated with reduced renal secretion of creatinine^18^, a key physiological side effect observed in AS mice^7,19^. Many other molecular carriers and lipid transport associated genes were modulated largely suggesting altered lipid metabolism in AS GEC (Fig. 7C, D). The heatmaps in Fig. 7F, G show the most significantly regulated genes involved in glucose metabolism in both GEC subpopulations in AS mice. Transcripts with loss of function, such as *Apom*, *Mc4r* and *Esr2* are strongly associated with glucose intolerance and/or insulin resistance. Glucose transporters, such as *Slc2a2* and *Slc2a4* were also downregulated suggesting a potential imbalance in glucose uptake and metabolic homeostasis. The dim^tdT^ cells showed a pattern of gene expression similar to that of the bright^tdT^ cells, thus indicating that energy metabolism of both subpopulations of GEC might be compromised (Fig. 7F–G).

### Endothelial glycocalyx-associated gene expression in WT and AS GEC

**Figure 8:**
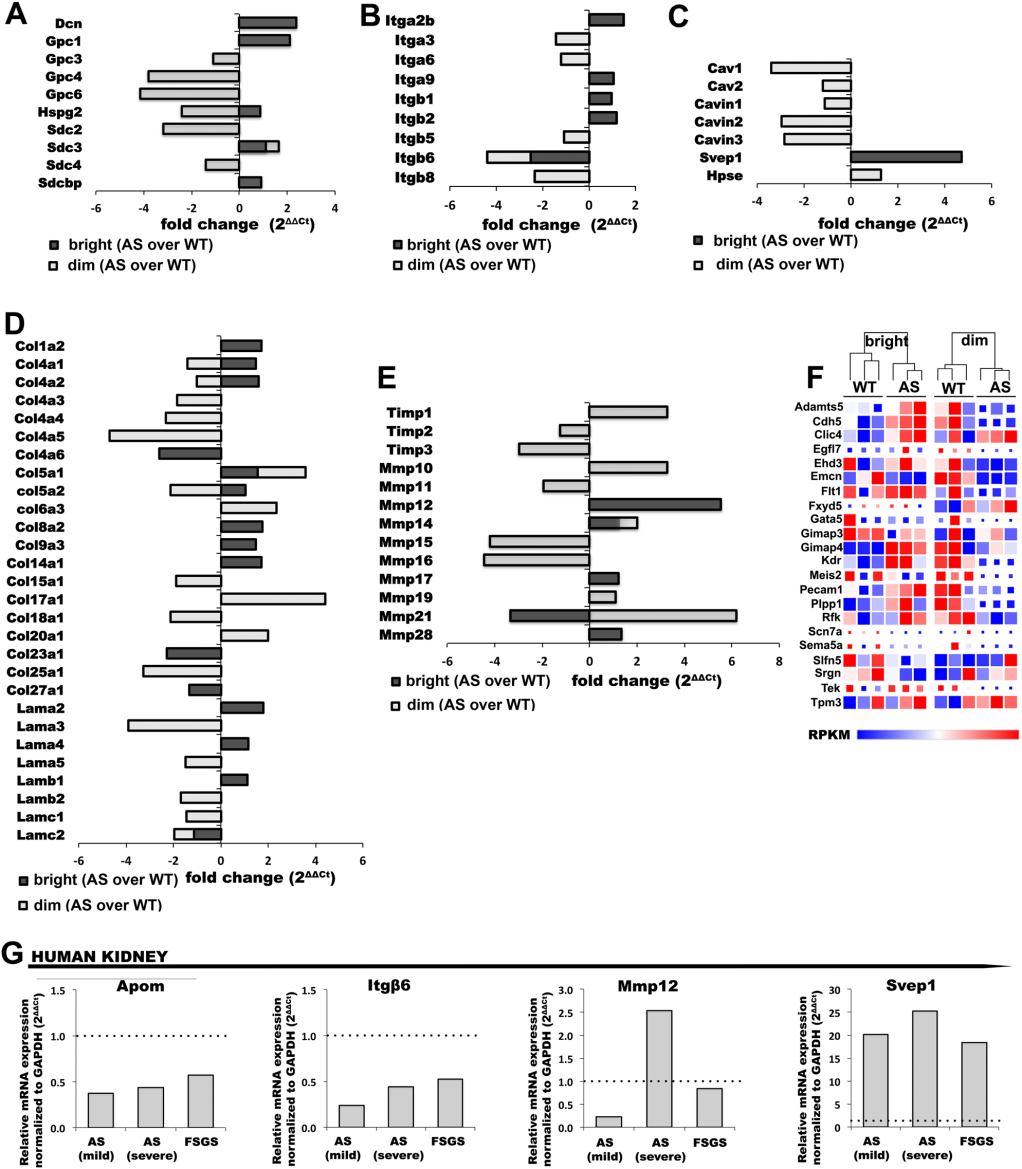
Comparison of extracellular matrix related differential gene expression in dim^tdT^ and bright^tdT^ subpopulations in AS over WT controls, and RT-qPCR analysis of Apom, Itgb6, Mmp12 and Svep1 expression in human kidney biopsy samples from Alport syndrome and FSGS patients**. (A–C)** Bar graphs summarizing the fold change regulation of genes expressed in bright^tdT^ (AS over WT – black bars) and dim^tdT^ (AS over WT – grey bars) subpopulations for glycocalyx associated proteins (**A**), integrins (**B**), extracellular matrix associated proteins (**C**). (**D, E)** Bar graphs showing the fold regulation of extracellular matrix proteins (**D**), Timps and Mmps (**E**) in bright^tdT^ (AS over WT—black bars) and dim^tdT^ (AS over WT—grey bars) subpopulations. (**F)** Heatmap diagrams with color-coded representation of the RPKM values comparing the transcriptional expression of endothelial specific genes for each subpopulation in AS to that of WT. The color scale represents relative gene expression levels across each row with red denoting upregulation and blue denoting downregulation. Color intensity indicates stronger regulation. (**G)** Bar graphs showing RT-qPCR analysis and relative expression of Apom, Itgβ6, Mmp12 and Svep1 gene expression in human kidney tissue samples from AS patients with low and high level of proteinuria (AS-mild patient (n = 1): 17 years male, proteinuria: 2.5 g/day; AS-severe patient (n = 1): 22 years male, proteinuria: 10 g/day) and a 35 years male FSGS patient (n = 1) with proteinuria of 3.2 g/day as compared to a heathy donor and normalized to GAPDH according to the 2^∆∆Ct^ method. (Biological replicated A-F, n = 3 mice/group).

Endothelial glycocalyx is a network of membrane-bound proteoglycans and glycoproteins, which serves as the primary layer of the glomerular filtration barrier and damage to its structure has been associated with various renal diseases^20,21^. Several glycocalyx-associated proteins were differentially regulated in the GEC subpopulations both in WT and AS mice, including decorin, (known to regulate TGF-beta levels)^22^ expressed in the bright^tdT^ cells only (Suppl. Fig. 5A). When compared to WT, the AS-dim^tdT^ and not the bright^tdT^ cells showed significant downregulation of *Sdc2, Gpc4 and Gpc6* (Fig. 8A), which might suggest new, previously unknown mechanisms of CKD progression in AS.

### Integrins, ECM and endothelial specific gene expression in WT and AS GEC

Integrins^23–25^ and ECM^26–28^ play indispensable role during renal development, provide integrity to the glomerular filtration barrier (including GEC) and contribute to fibrosis in CKD. Integrin expression in AS-GEC subpopulations was similar to that of WT (Suppl. Figure 5B). Instead, *Itgβ6* was highly downregulated in AS-bright^tdT^ and AS-dim^tdT^ relative to WT controls (Fig. 8B). *Cav1* and other caveolae associated proteins were downregulated in the AS-dim^tdT^, but not in the AS-bright^tdT^ GEC (Fig. 8C, Suppl. Figure 5C). In contrast, *Svep1*, which mediates Itg*α* 9β1-dependent cell adhesion^29^ was drastically upregulated in the AS-bright^tdT^ but not the AS-dim^tdT^ cells (Fig. 8C, Suppl. Figure 5C). Mutations in the *Col4α 5* produce GBM defects affecting multiple components of the ECM in AS mice. ECM protein expressions were higher in the bright^tdT^ cells both in WT and AS (Suppl. Figure 5D). In AS, expression of many collagens and laminins were drastically affected, such as *Col17α 1 and Lamα3 in the dim*^*tdT*^* cells* (Fig. 8D). Matrix metalloproteinases (MMPs), including MMP-12 and -14 in AS-bright^tdT^ and MMP-10,15,16 and MMP-21 in the AS-dim^tdT^ cells were similarly impacted (Fig. 8E), thus indicating possible shifts in the regulation of ECM homeostasis. Expressions of endothelial specific genes in AS-GEC subpopulations were also remarkably different compared to WT. Most transcripts in the bright^tdT^ were upregulated in AS relative to WT mice, while in the dim^tdT^ it was the opposite (Fig. 8F).

### Gene expression in kidneys from patients with AS and FSGS

We used kidney biopsy samples from patients with AS to establish whether some of the observed important gene modulations in our mouse model of AS are also detectable in the human form of the disease. Total cortical tissue available in the biopsy was used as compared to GEC only in the mouse model. We observed similar trend of gene expression for *Apom*, *Itgβ6*, *Mmp12* and *Svep1* in AS kidney compared to normal human tissue (Fig. 8G). Similar results were also observed in a sample from FSGS patient, thus suggesting that these genes might play an important role not only in AS but also in other forms of CKD.

## Discussion

Endothelial cell heterogeneity has been reported in macro- and microvascular beds of different organs^30,31^, including the kidney^32^ and human cancers^33^. In an elegant study, Barry et al. demonstrated that kidney vascular heterogeneity diversifies perinatally and throughout adulthood^32^. Recently, evidence for intraglomerular heterogeneity of endothelial cells has emerged. Dumas SJ et al. described five transcriptionally distinct subclusters of endothelial cells suggestive of their spatial position in the glomerular compartment by scRNA-seq in healthy mice, and their adaptation through differential gene expression when exposed to water deprivation^6^. In a similar approach Karaiskos N. et al. identified four different gene clusters of EC in healthy glomeruli with overrepresented genes in cell maturation, stress response, cell adhesion and endothelial proliferation^2^. Therefore, understanding the associations between GEC heterogeneity and AS by studying the change in gene expression in the diseased state could potentially identify new mechanisms of disease progression and/or identify novel targets for treatment. In our study, differently from the studies cited above, we performed bulk RNA-seq on endothelial cells isolated directly from purified glomeruli based on the tdTomato protein expression and analyzed changes in their transcriptome in AS versus healthy mice. This approach allowed us to not only obtain live cells but also characterize GEC heterogeneity based on protein expression rather than RNA and validate their phenotype by molecular biology techniques. In particular, in our mouse model of AS, we identified two distinct GEC subpopulations (dim^tdT^ and bright^tdT^), based on the magnitude of the *tdT* protein expression. Consistent with the sc-RNA sequencing data published by other groups^2,6^, analysis of the FACS tdT signal distributions and imaging studies of glomeruli by confocal and intravital microscopy suggest intraglomerular heterogeneity of EC.

We are aware that Cre driver should give a binary outcome (on-off) following lox-P mediated excision. However, Cre-lox expression systems are often influenced by complex biology, and often hard to interpret. We believe that in our mouse model, the low level of Tek in the dim^tdT^ cells translates into a fewer recombination events (fewer Cre molecules excising a small percentage of transgene integrants), resulting in two different fluorescence intensity signals from identical locus. Consistent with our findings, Bapst AM et al. demonstrated the presence of low and high intensity tdTomato expressing cells in the kidney from the identical promoter^34^.

A snapshot of the active transcriptome suggests significant cellular transformations within the GEC subpopulations. We have previously shown that GEC injury is an early event in AS pathology, characterized by de novo expression of PLVAP, increased VEGF signaling and decreased endothelial glycocalyx proteins, which precede the symptoms of heavy proteinuria^19^. The glycolalyx is a dynamic structure that undergoes constant remodeling to maintain homeostatic balance. Fragmentation and shedding of glycocalix might be triggered by an inflammatory response^35–38^, or predictive of development of kidney injury^39,40^; downregulation of its components in the dim^tdT^ cells might indicate homeostatic imbalance and be linked to similar cause and effect relationships in our model of AS.

GEC remodel and interact with the GBM principally via integrin receptors^41^. Loss of* Itgβ6*, (component of integrin αvβ6) could have significant impact on GEC interactions with LAP/TGF-*β* complex^42–44^ fibronectin^44^ and osteopontin^45^, all of which have important roles in the progression of CKD. Itgα9β1 upregulation, which mediates adhesion of endothelial cells through interactions with Vcam1^46^ and Svep1 (a high affinity ligand for integrin α9β1 ^47^, is consistent with the enrichment of this pathway in the bright^tdT^ cells. Moreover, Svep1 signaling via Itgα9β1 is suggested to contribute to lymphatic valve formation in mice^48^. Svep1 also regulates transcription factor Foxc2 in lymphatic endothelial cells possibly through the angiopoietin-2 and Tie1/Tie2 receptor system as a component of the lymphatic vessel remodeling mechanism^49^. Physiological function of Svep1 in GEC has not been established, however, its upregulation in AS along with increased expression of Itgα9β1 in the bright^tdT^ GEC is highly suggestive for similar remodeling function as described for the lymphatic vessels. In contrast, these mechanisms are relatively dormant in the dim^tdT^ cells.

Energy metabolism in endothelial cells plays pivotal role in various pathologies, including CKD^50^. The link between oxidative stress involving mitochondrial damage and endothelial dysfunction is well established in diabetic kidney disease^51^. The notion of dysfunctional endothelium in the progression of AS is a relatively new area of research. To the best of our knowledge, this study is the first to demonstrate that the glomerular endothelium in AS contains transcriptionally heterogeneous cells, which exhibit diverse gene profiles involved in mitochondrial function, glucose and lipid metabolism.

GEC directly interact with immune cells and circulating factors in the blood and crosstalk with podocytes and mesangial cells, therefore they could be potent intra-glomerular contributors to inflammatory processes. Yet, there are no data concerning GEC involvement in AS inflammation and production of pro-inflammatory mediators. Human umbilical vein endothelial cells^52,53^ and human brain microvascular endothelial cells^54^, have been shown to elicit inflammatory responses when stimulated with pro-inflammatory stimuli, such as TNF-α, IL-1β or LPS. In addition, pro-inflammatory stimulation of conditionally immortalized human GEC has been shown to lead to strong expression of inflammatory proteins, including VCAM-1 and ICAM-1^55^. Thus, it could be hypothesized that similar mechanisms of action could be occurring also in AS glomeruli. Our findings indicate that GEC (both dim^tdT^ and bright^tdT^) from AS mice are significantly enriched in genes and pathways involved in different aspects of immune responses (Suppl. Table 5–6). Apln/APJ system plays a variety of biological functions, including in kidney disease^56–62^, but its role in AS is unknown. Apelin upregulation in bright^tdT^ but not in dim^tdT^ GEC, and in AS patient kidney specimen underscore the importance of this signaling mechanism as a potent inflammatory mediator. In response to in vitro Apln-13 stimulation HUVECs release adhesion molecules, such as ICAM-1, VCAM-1 and MCP-1^63^. It took Apln-13 and pyr-Apln-13 isoforms together in the case with human GEC to generate similar response, indicating that different Apelin isoforms might be required for a pro-inflammatory activation of glomerular endothelial cells. In addition, there is data to suggest that MMP-12 induction might be linked to MCP-1-mediated activation of the CCR2 receptor, previously described in macrophages^64^ and podocytes of AS mice^65^. Abraham and colleagues demonstrated that MMP-12 deficiency reduces macrophage infiltration in both glomeruli and the interstitium and attenuates crescentic anti-GBM glomerulonephritis^66^, which ultimately supports the notion proposed by Liu and colleagues that MMP are not limited to digestion of matrix, but rather participate in all levels of renal pathologic process, including inflammation^67^.

We acknowledge several limitations in our study. First, bulk RNA-seq approach does not allow for characterization of GEC subclusters similar to that of the sc-RNA method. Nevertheless, using our tdT expression based-approach, we clearly identified two subtypes of GEC with distinct gene signatures in healthy mice, which were differently regulated in the diseased state in AS. We also recognize that our GEC characterization is performed at one time point (4-month, mild proteinuria). Evaluation of additional time points, for instance an earlier time point, will be informative of disease initiating molecular signaling mechanisms involved in the onset of AS pathogenesis that can be modulated to prevent renal progression.

In sum, this study provides a novel insight into GEC transcriptional changes into a model of CKD, AS. Data suggest that in chronic pro-inflammatory and pro-fibrotic conditions of AS, GEC subpopulations could take overlapping and diverse roles contributing to the inflammatory and metabolic dysfunction of the glomerular endothelium. Data from human AS and FSGS samples provide important validation of our findings in the mouse model of AS. Insight gained from the present study could advance our understanding of the inner workings of the glomerular microvasculature, its potential role in pro-inflammatory processes, and facilitate the identification of new therapeutic targets for intervention.

## Methods

### Animal models

AS Tek^tdT^ mice were generated by breeding AS mice (B6.Cg-Col4α5tm1Yseg/J) with an endothelial specific Cre-driver mouse (B6.Cg-Tg(Tek-cre)1Ywa/J and a tdTomato-reporter mouse (B6.Cg-Gt(ROSA)26Sor^tm14(Cag-td-Tomato)Hze^/J); these mice express tdTomato (*tdT*) in all endothelial cells including GEC. All mouse strains were obtained from Jackson Laboratories. Animal studies were performed in accordance with guidelines approved by the Institutional Animal Care and Use Committee at the Children’s Hospital Los Angeles. A total of 33 WT-Tek^*tdT*^ and 29 AS-Tek^*tdT*^ mice were used in our studies.

### Proteinuria measurement

Urine samples were collected overnight using metabolic cages (Harvard Apparatus #PY8 72–9,061) once every four weeks, starting when mice were 1 month old and completed when they reached 6 months of age. The urine albumin-to-creatinine ratio was determined by ELISA for albuminuria (Immunology Consultants Laboratory # E90AL), and quantitative colorimetric assay kit for urine creatinine was performed as published^7,19^.

### Glomerular digestion and GEC isolation by FACS

To obtain GEC, renal cortices from WT-Tek^tdT^ and Alport-Tek^tdT^ were isolated and mechanically minced for 5′ on ice followed by enzymatic digestion with 1% collagenase type I (Worthington) solution prepared in RPMI-1640 (Gibco) for 30′ at 37 °C. Tissue lysates were passed through a 100 μm then 40 μm nylon mesh strainers (Corning Inc., MA) and washed several times with saline solution (PBS, ThermoFisher Scientific, MA). Glomeruli harvested from the 40 μm mesh were further digested with 0.25% TrypLE (ThermoFisher Scientific, MA) solution supplemented with 0.6% collagenase IV (Worthington) for 20′ at 37 °C to obtain single cells. Cells were then passed through a 100 μm strainer again to remove any clumps. To sort the GEC for tdT expression, single cells were suspended in 1 × PBS buffer and sorted using FACSAria III sorter (DB Biosciences, CA). Gating strategy to remove debris and doublets were applied as presented in Suppl. Figure 6A–D. Positively sorted cells were collected into 1.5 mL eppendorf tubes filled with complete endothelial culture media (Cell Biologics Inc., IL), centrifuged for 5′ at 200 g and analyzed as proposed for different experiments.

### Flow cytometry and analysis

Flow cytometric analysis of mouse tdT GEC, and human GEC was performed using a BD FACSCanto II (DB Biosciences, CA). Data acquisition and analysis of samples were performed using the BD FACSDiva 5.1.3 and FlowJo 10.5.3 software. Briefly, to quantify Ehd3 and WT1 expression in mouse tdT GEC, the cells were stained with Zenon AF488-conjugated anti-Ehd3 and AF647-conjugated anti-WT1 antibodies according to manufacturer instruction (ThermoFisher Scientific, MA), and using gating strategy presented in Suppl. Figure 6E–J. Human GEC were stained with Zenon AF488-conjugated anti-Ehd3 antibody. Gating strategies shown in Suppl. Figure 6K–L were applied for the analysis. Antibody concentrations are reported in Suppl. Table 8.

### Immunofluorescence, confocal and multiphoton microscopy, and morphometric quantification of tdT positive cells

Thin deparaffinized kidney Sects. (5 μm) were blocked in 5% BSA and immunostained for fluorescence microscopy with antibodies applied overnight at 4 °C (Suppl. Table 8). Alexa Fluor-conjugated secondary antibodies (ThermoFisher Scientific, MA) were applied at 1:500 dilution with 30′ incubation at room temperature. A Leica DM RA fluorescent microscope was used in conjunction with Open Lab 3.1.5 software to image the staining. Confocal z-stack images of intact glomeruli were obtained with an LSM 700 system mounted on an AxioObserver.Z1 inverted microscope equipped with a C-Apochromat 40x/1.20 water-immersion lens. Time lapse images of live cells were obtained with an AxioObserver.Z1 inverted microscope equipped with an Axiocam 702 camera and an environmental chamber to maintain the culture at 37** °C** and 5% CO2 (Carl Zeiss Microscopy, Thornwood, NY). Videos were generated from z-stack and time lapse images with FIJI ImageJ software ^68^. Under continuous anesthesia (Isoflurane 1–4% inhalant via nose-cone), Tek^tdT^ mice, in which the left kidney was exteriorized through a flank incision, were placed on the stage of the inverted microscope with the exposed kidney placed in a coverslip-bottomed chamber bathed in normal saline as described previously^69^. Body temperature was maintained with a homeothermic blanket system (Harvard Apparatus). Alexa Fluor 680-conjugated bovine serum albumin (Thermo Fisher, Waltham, MA) was administered iv. by retro-orbital injections to label the circulating plasma (30 µL iv. bolus from 10 µg/ml stock solution). The images were acquired using a Leica SP8 DIVE multiphoton confocal fluorescence imaging system with a 63 × Leica glycerine-immersion objective (numerical aperture (NA) 1.3) powered by a Chameleon Discovery laser at 960 nm (Coherent, Santa Clara, CA) and a DMI8 inverted microscope’s external Leica 4Tune spectral hybrid detectors (emission at 550–650 nm for tdTomato and 675–750 nm for Alexa Fluor 680) (Leica Microsystems, Heidelberg, Germany). The potential toxicity of laser excitation and fluorescence to the cells was minimized by using a low laser power and high scan speeds to keep total laser exposure as minimal as possible. The usual image acquisition (12-bit, 512 × 512 pixel) consisted of only one z-stack per glomerulus (< 3 min), which resulted in no apparent cell injury. Fluorescence intensity measurements were performed in time-lapse (xyz) mode in multiple glomeruli. Image analysis and fluorescence intensity measurements were assessed by LAS X software (3.3.0.16799), Leica Microsystems). To perform morphometric quantification of tdT positive cells, tdT signal intensity in the optical sections of an intact kidney were analyzed on a LasX program (Leica) using a double-blinded method. Z-stack images of randomly imaged glomeruli (n = 8) from two different mice, were selected for analysis. Individual cells and cell boundaries (n = 87) were identified by simultaneously viewing 3 orthogonal cross-sections (XY-, YZ- and XZ- planes) for each glomerulus. ROIs of 1.5 µm in diameter were placed on each selected cell such that the maximum tdT signal intensity in the cell was the highest possible to make sure the signal was measured at its peak. Signal intensity values were displayed as a line graph relative to the z-position of the image (Suppl. Figure 7). Peaks that were plateaus as opposed to bell-shaped curves were omitted from further analysis. Peaks within a 15 µm range of each other were selected for further analysis, excluding any peaks deeper than 35 µm. The 3 highest values of each ROI were then used to arrange the ROIs in order of decreasing peak depth. The boundary between cells that were considered “bright” and “dim” was visually evident and it was established such that the difference between the lowest peak of the “bright” group and the highest peak of the “dim” group was greater than the difference between peaks within each group. This process was repeated for each glomerulus. The maximum value of each ROI was then taken for further analysis. A Welch’s T-test (two-tailed, unpaired) was applied to determine statistically significant differences between the average values of bright^tdT^ and dim^tdT^ cells per glomerulus.

### Real-Time PCR and Western blot

Total RNA from experimental groups was extracted using the Qiagen RNeasy Micro kit as per manufacturer recommendations. After cDNA production, quantitative Real-Time PCR for Tek and CD133 was carried out using a Roche Light Cycler 480 and KAPA SYBR FAST qPCR Master Mix (Kapa Biosystems, MA) according to manufacturer’s instructions (Suppl. Table 9). Real-Time PCR conditions were as follows: 90 °C for 10′, 60 °C for 10″, and 72 °C for 1″ with the analysis of the fluorescent emission at 72 °C. Forty cycles were performed for each experiment.

To evaluate the expression of Apelin, Apom, Itgβ6, Mmp12 and Svep1 from human biopsy samples of AS and FSGS, total RNA was extracted from paraffin embedded tissue slides using FFPE RNA Purification Kit (Norgen) following manufacture’s instructions. cDNA was pre-amplified for 14 cycles, followed by Real-Time PCR as previously described. Pre-amplification conditions were as follows: 94 °C for 10′, 94 °C for 15″ and 60 °C for 4′ (14 cycles), 99 °C for 10′ and held at 4 °C.

Total protein from the experimental groups were collected and stored at − 80 °C in a RIPPA assay buffer supplemented with protease and phosphatase inhibitors (ThermoFischer Scientific) until use. Protein electrophoresis was performed on 4–20% Tris–Glycine gels and transferred onto a polyvinylidene fluoride 0.45-μm membrane (Millipore) and probed with antibodies with overnight incubation at 4 °C (Suppl. Table 8) for the list of antibodies and specific concentrations). HRP-conjugated secondary anti-rabbit antibodies (Sigma-Aldrich) were applied it 1:20,000 ratio. Antigens were detected using the ECL Western Blotting detection reagents (Amersham Biosciences/GE Healthcare), impressed on Biomax Light Film (GE Healthcare) and developed on Konica SRX101A film processor. Data from 3 independent experiments were quantified by densitometry using image J (NIH) (all measurements were normalized against their corresponding housekeeping gene, β-actin) and further processed with photoshop (Adobe photoshop CC 20.0.9 Release).

### RNA extraction, sequencing and data analysis methodology

RNA extraction from tdT positive GEC subpopulations from WT (n = 3) and AS (n = 3) mice was performed immediately after FACS as above. The RNA integrity was checked by Agilent Bioanalyzer 2100 and samples with clean rRNA peak (RIN > 7) were used for further experiments. Library for RNA-seq was prepared according to KAPA Stranded mRNA-seq poly-A selected kit with 200-300bp insert size (KAPA Biosystems, Wilmington, MA) using 250 ng total RNAs as input. Final library quality and quantity were analyzed by Agilent Bioanalyzer 2100 and life Technologies Qubit 3.0 Fluorometer. 150 bp PE (paired-end) reads were sequenced on Illumina HiSeq 4000 (Illumina Inc., San Diego, CA). Data processing was performed using the USC high performance-computing cluster (https://hpcc.usc.edu/). Roughly 50 million 150 bp paired-end sequences were aligned to the Gencode M16 annotation^70^ based on Genome Reference Consortium mouse genome (GRCm38.p5) using the STAR aligner with ‘GeneCounts’ output ^71^. Differential gene expression was determined using the R/Bioconductor software, ‘edgeR’^72^.

Gene set enrichment analysis was performed with the R/Bioconductor software ‘GOstats’^73^ using the Gene Ontology database (TGOC, 2017). Venn Diagrams based on the data were generated using the R/Bioconductor software package ‘Limma’^74^, and using the following cut offs: p < 0.05, logCPM > 1, and logFC > 1.5. Subsets taken from the Venn diagrams were used for enrichment analysis with the ‘GOstats’ software package. Directed acyclic graphs of GO terms were generated by ‘GOstats’ and subsets were imported into the Cytoscape software^75^ using the ‘RCy3′ software package^76^. Clustering and plotting of heatmaps was performed with the Morpheus versatile matrix visualization and analysis software (https://software.broadinstitute.org/ morpheus). Gene network graphs and pathway activation analysis were further performed with Ingenuity Pathway Analysis (IPA) software (Qiagen, MD).

RNA from the tissue was obtained from paraffin slides using FFPE RNA Purification Kit (Norgen) and RT-qPCR was performed as described.

### Ethics statement, acquisition of human samples, and cell culture of human cells

Kidneys deemed non-suitable for transplantation were used for isolation of human primary GEC and provided by Novabiosis (Promethera Biosciences Group). CHLA Institutional Review Board approved tissue collection. Discarded kidneys were harvested from infant patients with a non-nephrological cause of death, and thus our isolation of primary GEC rendered functional cell type.

De-identified tissue biopsies from healthy subject (n = 1), from individuals with AS (n = 2) or FSGS (n = 1) were obtained from the biorepository of S. Orsola-Malpighi Hospital, University of Bologna, Bologna, Italy. The Institutional Review Board of University of Bologna, Italy approved the protocols for the collection of these human samples. Informed consent for kidney donation and kidney biopsies was obtained from all participants. All experiments were performed in accordance with ethical guidelines and regulations of the Declaration of Helsinki.

To obtain human glomerular endothelial cells (hGEC), glomeruli were isolated by the sieving method as described above and cultured for 3–5 days on gelatin coated tissue culture dishes in Complete Human Endothelial Cell Media (CHECM) supplemented with 0.1% VEGF, 0.1% heparin, 0.1% EGF, 0.1% FGF, 0.1% hydrocortisone, 1% L-glutamine, 2% endothelial cell supplement, 10% FBS and 1% antibiotic–antimycotic solution (CellBiologics, IL). Primary cultured glomerular cells were prepared for cell sorting using standard techniques. Cells were labeled with human specific anti-CD31 antibodies conjugated to magnetic microbeads (Suppl. Table 8) and hGEC were sorted by autoMACS (Miltenyi Biotech, CA). Isolated cells were cultured on tissue culture flasks using the same method described above. The human neuronal stem cell line (HB1.F3.CD), was grown on tissue culture dishes as monolayer in DMEM supplemented with 10% FBS, 1% 2 mM L-glutamine and 0.25% primocin.

### Dil-Ac-LDL update assay

hGEC and HB1.F3.CD cultures were prepared for the DiI-Ac-LDL assay (Cell Applications Inc) as per manufacturer’s instructions. Cultures of hGEC and HB1.F3.CD were plated into 4-well tissue chamber slides (for hGEC pre-coated with Extracellular Matrix Attachment Solution; Cell Applications Inc) at a density of 6.0 × 10^4^ cells per well and allowed to grow to about 95% confluency. The media was removed and 10 µL of DiI-Ac-LDL was added to the cells in 200 µL of fresh CHECM (for hGEC) and DMEM (for HB1.F3.CD) incubated for 4 h at 37 °C, 5% CO_2_ incubator. The slides were mounted and internalization and degradation of DiI-AC-LDL was assessed at 552 nm of excitation (red fluorescence) by inverted fluorescence microscope (Leica DMI6000 B).

### In vitro Apelin stimulation assay

hGEC were plated into 6-well tissue culture dishes pre-coated with gelatin-coated solution at a density of 2.0 × 10^5^ cells per well and allowed to grow overnight. Apelin-13, *pyr*-Apelin-13 or combinations of both were added to the cells at a concentration of 1 × 10^−7^ M in CHECM medium. Cells not treated were used as control. The results of Apelin stimulations were assessed at 24 and 48 h by Western blot. All in vitro experiments were repeated in triplicate.

### Statistical analysis

Transcriptomic studies of GEC were performed using three biological replicates per group. Statistical analyses for imaging studies, PCR and western blot data were performed using R Studio (RStudio, MA), and Prism 8 (GraphPad Software, CA) software. Statistical differences between multiple groups were determined using One-way ANOVA, between two groups an unpaired *t-*test. A * p* value of less than 0.05 was considered as statistically significant. Data are shown as mean ± SD, unless otherwise noted.

## Supplementary information

Supplementary table 1

Supplementary table 2

Supplementary table 3

Supplementary table 4

Supplementary table 5

Supplementary table 6

Supplementary table 7

Supplementary table 8

Supplementary table 9

Supplementary figures

Supplementary video 1

Supplementary video 2

## Data Availability

The data supporting the findings of this study are openly available in Gene Expression Omnibus (GEO) under the accession number GEO: GSE135442 at the following link: https://www.ncbi.nlm.nih.gov/geo/query/acc.cgi?acc=GSE135442
