# Supplementary material for: Glomerular endothelial cell heterogeneity in Alport syndrome
Source: Sci Rep. 2020 Jul 10;10:11414. doi: 10.1038/s41598-020-67588-0 (PMC7351764; doi:10.1038/s41598-020-67588-0)

Glomerular Endothelial Cell Heterogeneity in Alport Syndrome

Hasmik Soloyan, MD[1], Matthew Thornton, MS[2], Valentina Villani, PhD[1], Patrick Khatchadourian, BS[1], Paolo Cravedi MD[3], Andrea Angeletti MD[4], Brendan Grubbs MD[2], Roger De Filippo, MD[1,5], Laura Perin, PhD[1,5], Sargis Sedrakyan, PhD[1,5]

1. GOFARR Laboratory for Organ Regenerative Research and Cell Therapeutics in Urology, Children's Hospital Los Angeles, Division of Urology, Saban Research Institute, University of Southern California, Los Angeles, USA.
2. Maternal Fetal Medicine Division, University of Southern California, Los Angeles, USA.
3. Division of Nephrology, Department of Medicine, Icahn School of Medicine at Mount Sinai, New York, New York
4. Nephrology Dialysis and Renal Transplantation Unit, S. Orsola University Hospital, Bologna, Italy.
5. Department of Urology, Keck School of Medicine, University of Southern California, Los Angeles, USA.

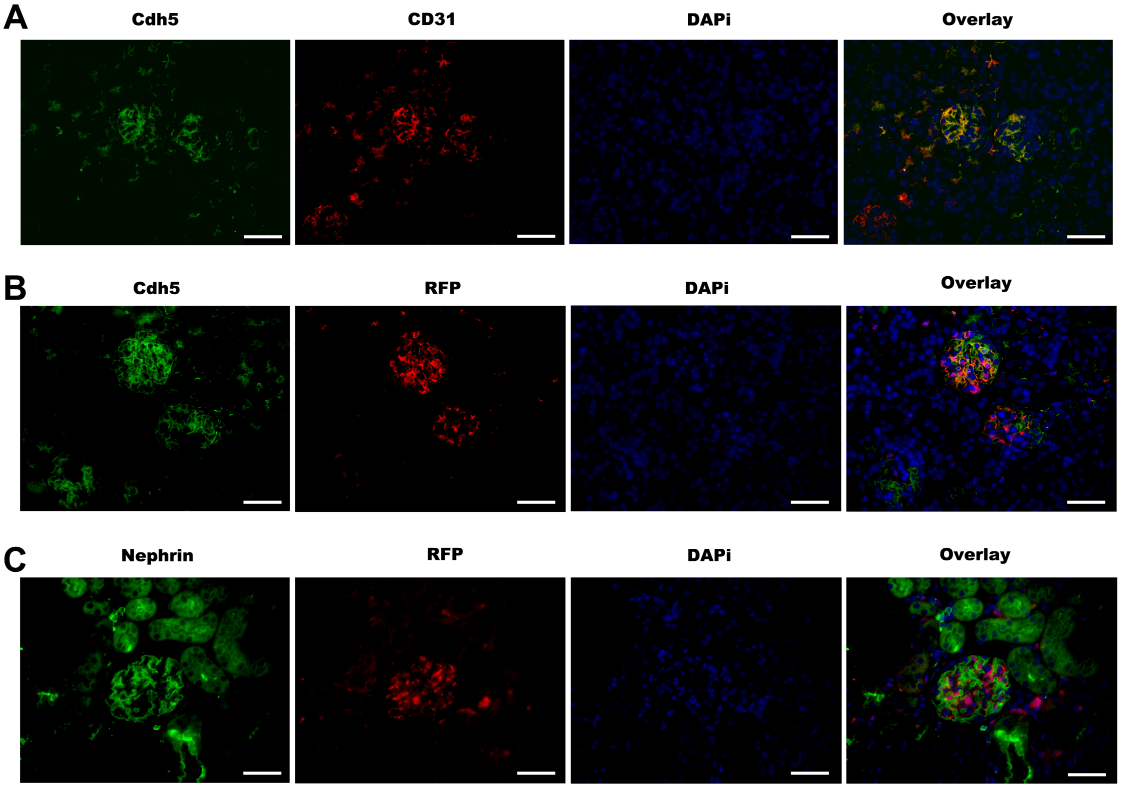

Supplementary Figure 1: Histologic evaluation of the tdTomato specificity in the endothelial cells

A-C. Representative immunohistochemistry images of WT kidney glomeruli co-labeled for Cdh5 and CD31, Cdh5 and RFP, and nephrin and RFP shows the localization of the RFP (for detection of tdTomato) with the endothelial markers (CD31, Chd5) (A-B), but not with podocyte marker nephrin (C).

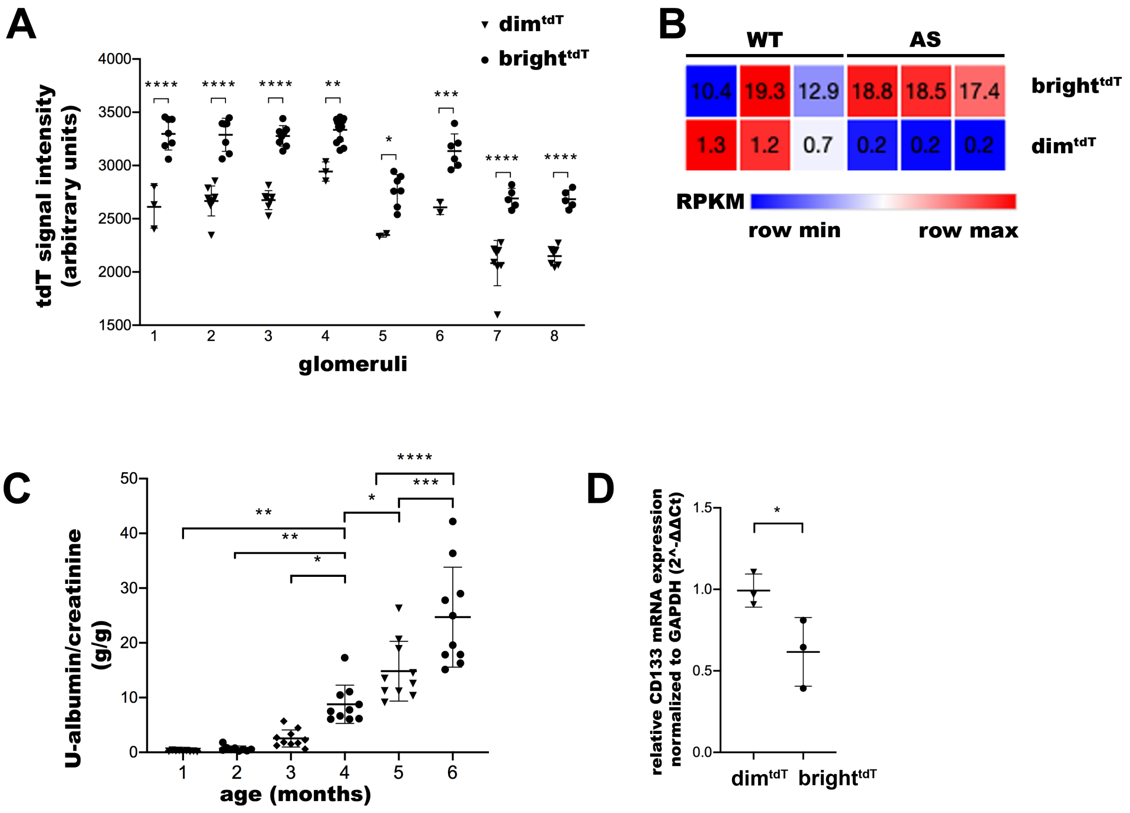

Supplementary Figure 2: Characterization of the tdT signal in the glomeruli

A. Dot plots showing quantification of tdT-signal intensity from individual glomeruli (n=8) acquired using a Leica SP8 DIVE multiphoton confocal fluorescence imaging system (also see Figure 1F). **B.** A heatmap diagram with color-coded representation of the RPKM values for Tek expression comparing the bright^{tdT} and dim^{tdT} GEC in WT, and differences in each subpopulation between AS and WT. The color scale represents relative gene expression levels across each row with red denoting upregulation and blue denoting downregulation of Tek. Color intensity indicates stronger regulation; (biological replicates, n=3/group) **C.** Dot plots showing proteinuria levels in AS mice from 1 to 6 month of age; (biological replicates, n=10). **D.** Dot plots showing RT-qPCR analysis of CD133 expression from dim^{tdT} and bright^{tdT} GEC normalized to GAPDH according to the 2ΔΔCt method; (biological replicates, n=3/group). The data are presented as mean ± SD. * denotes p-value < 0.05; ** denotes p-value <0.01; *** denotes p-value < 0.001 ; **** denotes p-value < 0.0001.

A

Genes	bright^{tdT} (AS over WT) UP	dim^{tdT} (AS over WT) DOWN
Bcl6b	1.042	-1.440
Fblim1	1.335	-3.607
Nphs1	1.758	-1.693
Ppfibp1	1.025	-1.481
Cd34	1.194	-2.167
Scrn1	1.109	-5.911
Wif1	3.524	-1.928
Adcy1	1.314	-1.705
Mrc2	1.203	-2.350
Ntn1	1.401	-3.206
Oplah	1.229	-1.927
Adcy5	2.067	-2.148
Arhgap28	1.611	-1.341
Myom1	1.387	-2.975
Maged2	1.178	-1.360
Col5a2	1.026	-2.127
Tyro3	2.369	-1.990
Lpin3	1.272	-7.501
Atp9a	1.006	-2.528
Ggt7	1.763	-1.820
Gucy1b3	1.060	-2.367
Cptp	1.150	-2.476
Nsg1	1.735	-4.148
Add2	1.408	-4.220
Lrig1	1.671	-1.580
Mfge8	1.005	-1.069
Col4a1	1.476	-1.428
Col4a2	1.605	-1.034
Ces2e	1.089	-3.069
Mcam	1.003	-1.415
Map3k13	1.551	-2.190
Loxl2	1.915	-3.898
Plvap	1.313	-1.863
Egln3	2.388	-2.059
Adamtsl2	1.115	-2.722
Tspan15	1.109	-1.897
Pcp4l1	1.127	-3.438
Cul7	1.234	-1.295
Bace2	1.030	-2.725
Dok4	1.161	-1.324
Ulk4	1.382	-2.392
Cldn5	1.102	-2.698
Pla2g6	1.363	-1.859
Ackr3	1.154	-3.531
Tcf21	1.190	-1.578
Cadm4	1.179	-1.478
Ntrk3	1.077	-1.411
Fat1	1.048	-1.364
Ccnd1	1.081	-1.313
Rgma	1.395	-2.942
Nt5dc2	1.389	-2.119
Rdh1	2.226	-1.975
Foxl1	1.807	-2.213

B

Genes	bright^{tdT} (AS over WT) DOWN	dim^{tdT} (AS over WT) UP
Zfp932	-1.483	1.443
Zfp182	-1.294	1.145
Stx19	-2.175	2.123
Serpinf1	-1.981	2.719
Rhou	-2.335	1.554
Plet1	-1.634	1.781
Nudt1	-1.652	1.422
Nrg1	-3.470	2.286
Gria3	-5.376	2.180
Gbp8	-1.722	1.143
Ccdc88c	-1.364	1.103
Bcl11b	-2.878	1.525
Arg1	-3.041	2.016

C

GO term	Pathway	Fold enrichment
GO:0001525	angiogenesis	10.1
GO:0043062	extracellular structure organization	9.98
GO:0098609	cell-cell adhesion	9.36
GO:0048514	blood vessel morphogenesis	8.49
GO:0032535	regulation of cellular component size	7.86
GO:0001568	blood vessel development	6.82
GO:0007155	cell adhesion	6.79
GO:0022610	biological adhesion	6.71
GO:0001944	vasculature development	6.49
GO:0090066	regulation of anatomical structure size	6.38
GO:0072358	cardiovascular system development	6.33
GO:0035239	tube morphogenesis	6.33
GO:0035295	tube development	5.89
GO:0072359	circulatory system development	5.49

Supplementary Figure 3: Inverse gene upregulations and downregulations between bright^{tdT} and dim^{tdT} GEC in WT and AS mice

A-B. Charts showing the fold-regulations of the 53 genes, upregulated in the bright^{tdT} and downregulated in the dim^{tdT} cells (A) and 13 genes, downregulated in the bright^{tdT} and upregulated in the dim^{tdT} cells (B), as depicted in the Venn diagram in Fig.5B. **C.** Pathway enrichment analysis of the 53 genes with respective GO terms from the same Venn diagram (C). Biological replicates, n=3/group

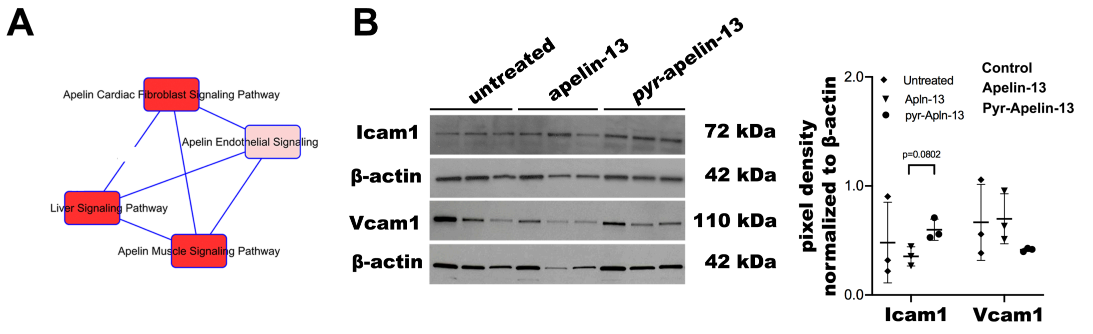

Supplementary Figure 4: Two isoforms of apelin (apelin-13 and pyr-apelin-13) fail to stimulate VCAM1 and ICAM1 expression in primary human GEC

A. A network diagram showing the top rated canonical pathways highlighting the strong regulation of Apelin/APJ (apelin receptor) signaling mechanisms as generated by ingenuity pathway analysis on the relative expression of AS bright^{tdT} vs WT bright^{tdT} GEC (color scale; red denotes strong upregulation, pink denotes weak upregulation). **B.** Human primary GEC were treated with 10^{-7}M apelin-13 or *pyr*-apelin-13 for 24hrs or were left untreated (control). Representative immunoblots comparing protein expression for VCAM1 (110 kDa) and ICAM1 (72 kDa) treated human GEC relative to untreated controls normalized against β-actin (42 kDa). Densitometric analysis of the immunoblots is shown in dot plots as pixel density measurements. Biological replicates, n=3/group. The data are presented as mean ± SD.

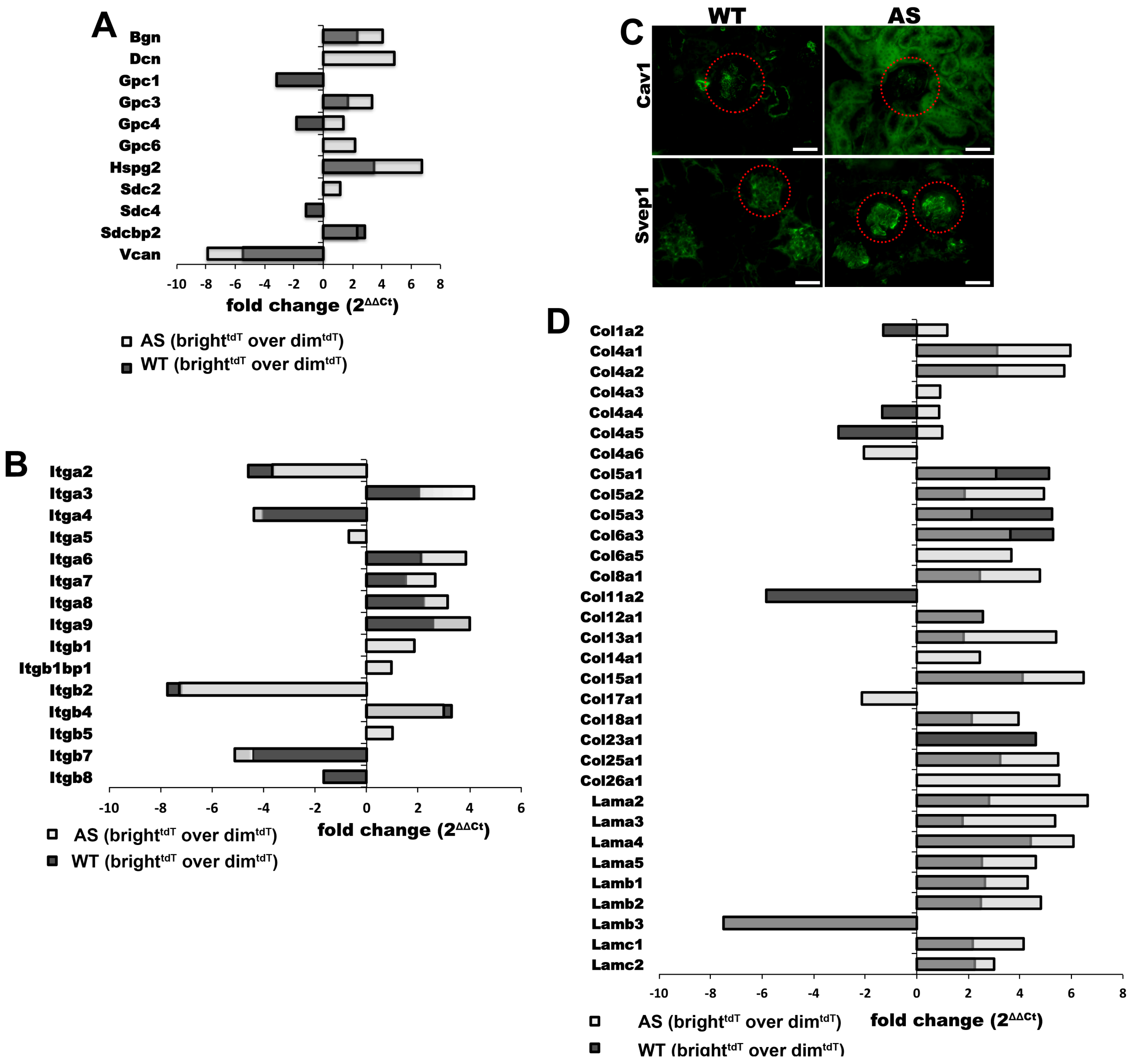

Supplementary Figure 5: Differential gene expression profiles between bright^{tdT} and dim^{tdT} GEC in WT and AS mice.

A-B. Bar graphs showing the fold change regulation of genes ($p<0.05$) expressed in AS (bright^{tdT} over dim^{tdT}, grey bars) and in WT (bright^{tdT} over dim^{tdT}, black bars) GEC for glycocalyx associated proteins (A), and integrins (B). **C.** Fluorescence images of WT and AS glomerular sections stained for Cav1 (AF488, green) and Svep1 (AF488, green). Dotted red circular lines highlight the glomeruli. Significant background green fluorescence signal in the tubules is evident. Scale bar = 50µm. **D.** Bar graphs showing the fold change regulation of genes ($p<0.05$) expressed in AS (bright^{tdT} over dim^{tdT}, grey bars) and in WT (bright^{tdT} over dim^{tdT}, black bars) GEC for extracellular matrix related proteins. Biological replicates for A,B,D, n=3/group.

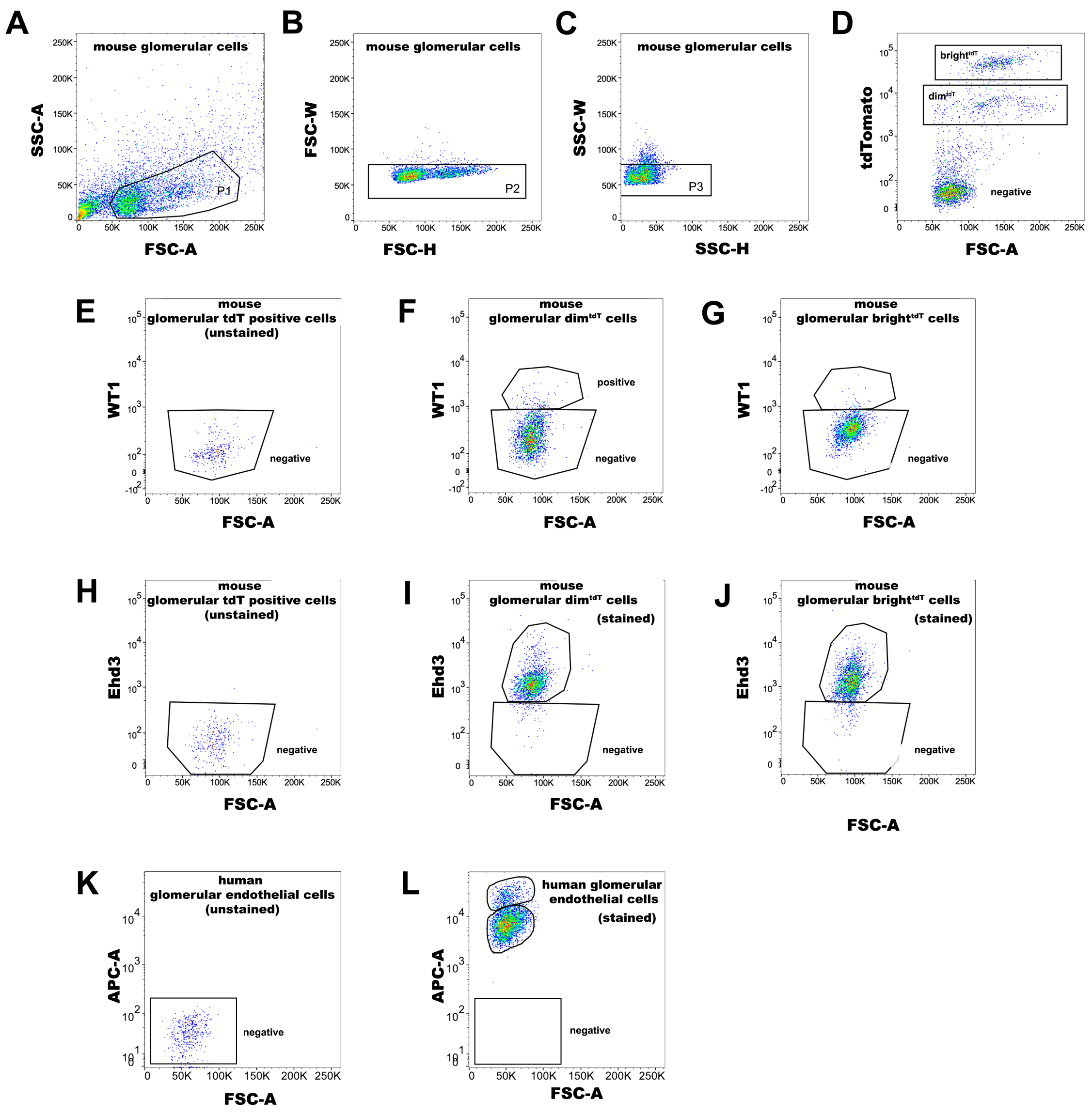

Supplementary Figure 6: Gating strategy for the flow cytometry characterization and isolation of the glomerular endothelial cells.

A-D. To characterize the GEC subpopulations, the cells were first gated on size (A) and singularity (B-C) followed by the gating for the two different intensities of the tdTomato signal (bright^{tdT} and dim^{tdT}) (D). To confirm the endothelial origin of the sorted dim- and bright^{tdT} cells, they were each co-immunostained for WT 1 (podocyte marker) and Ehd3 (endothelial marker). **E-L.** Gating for the bright^{tdT} and dim^{tdT} cells co-immunostained for WT 1 (podocyte marker) and Ehd3 (endothelial marker) were performed as follows: gates for the negative control for WT1 (E), and positive staining for WT1 in the dim^{tdT} (F) and bright^{tdT} GEC (G); gates for the negative

control for Ehd3 (H) and positive staining for Ehd3 in the dim^{tdT} (I) and bright^{tdT} GEC (J). Gating of an unstained primary human GEC as a negative control (K) and cells immunostained for Ehd3 (endothelial marker) (L).

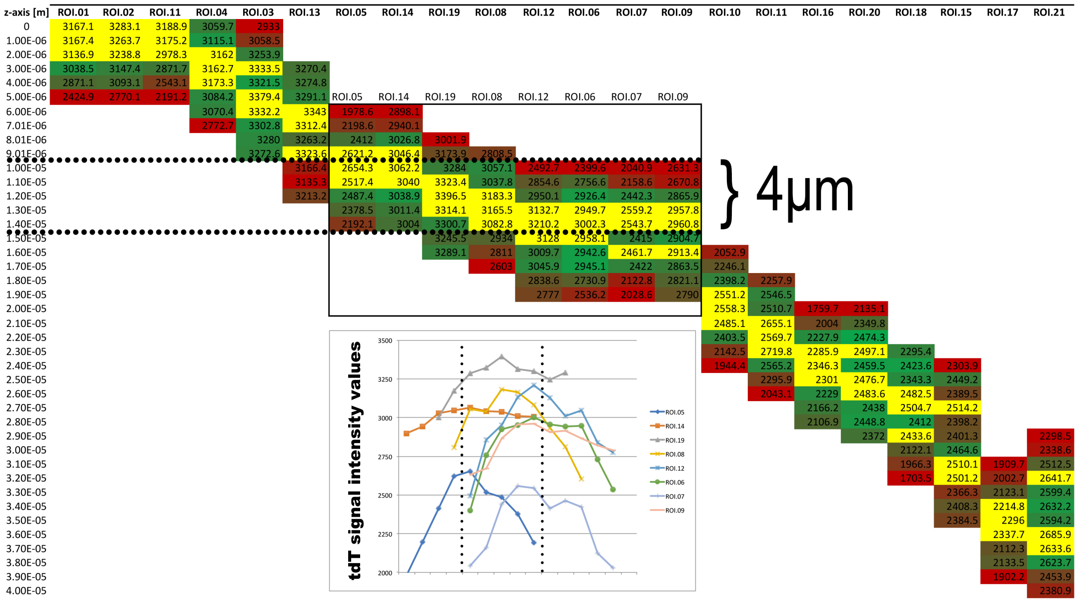

Supplementary Figure 7: tdT signal intensity assessment strategy

A representative raw data image illustrating the methodology used for the tdT signal analysis for each glomerulus. First, individual cells and cell boundaries were identified by simultaneously viewing 3 orthogonal cross-sections (XY-, YZ- and XZ- planes) for each glomerulus. tdT signal intensity values were obtained for each ROI and entered in a spreadsheet. The 3 highest values of each ROI were then used to arrange the ROIs in order of decreasing peak depth (shown above in yellow). Signal intensity values were displayed as a line graph relative to the z-axis. Peaks only within 4μm range of each other were used for final processing to eliminate any technical bias, and peaks deeper than 35μm were eliminated for the same reason. This process was repeated for each glomerulus. The maximum value of each ROI was then taken for further analysis.

Full-length images of the Western blot data with annotations

Fig. 2E: Western blot image showing protein expression for Tek/Tie2 and beta actin in the WT dim^{tdT} and bright^{tdT} GEC.
The first two lanes (labeled p8, non GEC) are unrelated to Fig. 2E. They are <u>none</u> dim^{tdT} and <u>none</u> bright^{tdT} cells.

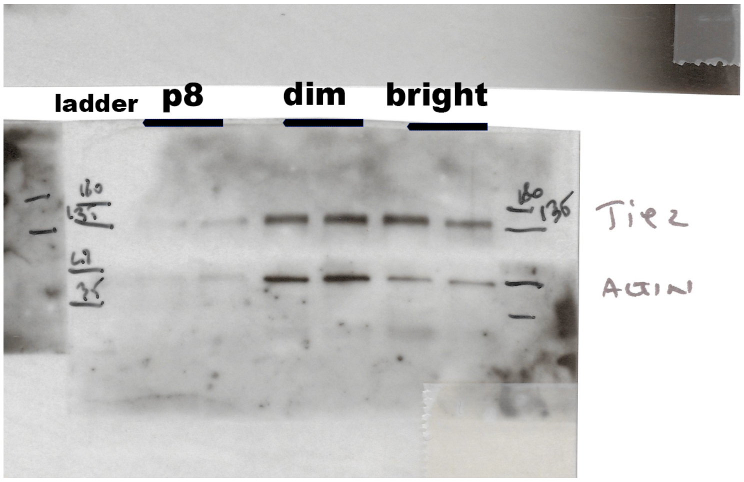

Fig. 2E: Western blot image showing protein expression for Ehd3 in the WT dim^{tdT} and bright^{tdT} GEC
The first two lanes (labeled p8, non GEC) are unrelated to Fig. 2E. They are <u>none</u> dim^{tdT} and <u>none</u> bright^{tdT} cells.

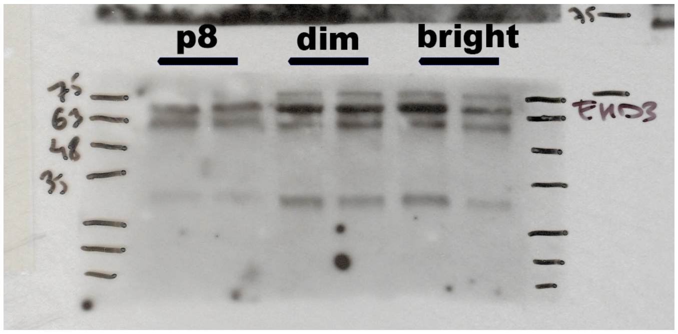

Fig. 2E: Western blot image showing protein expression for Cdh/Ve-cad in the WT dim^{tdT} and bright^{tdT} GEC

The first two lanes (labeled p8, non GEC) are unrelated to Fig. 2E. They are <u>none</u> dim^{tdT} and <u>none</u> bright^{tdT} cells.

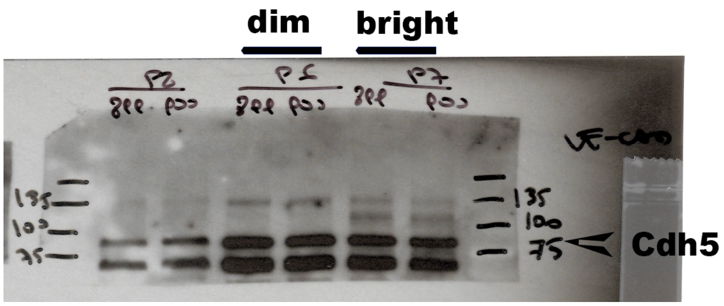

Fig. 6D: Western blot image showing protein expression for Apelin in WT and AS GEC

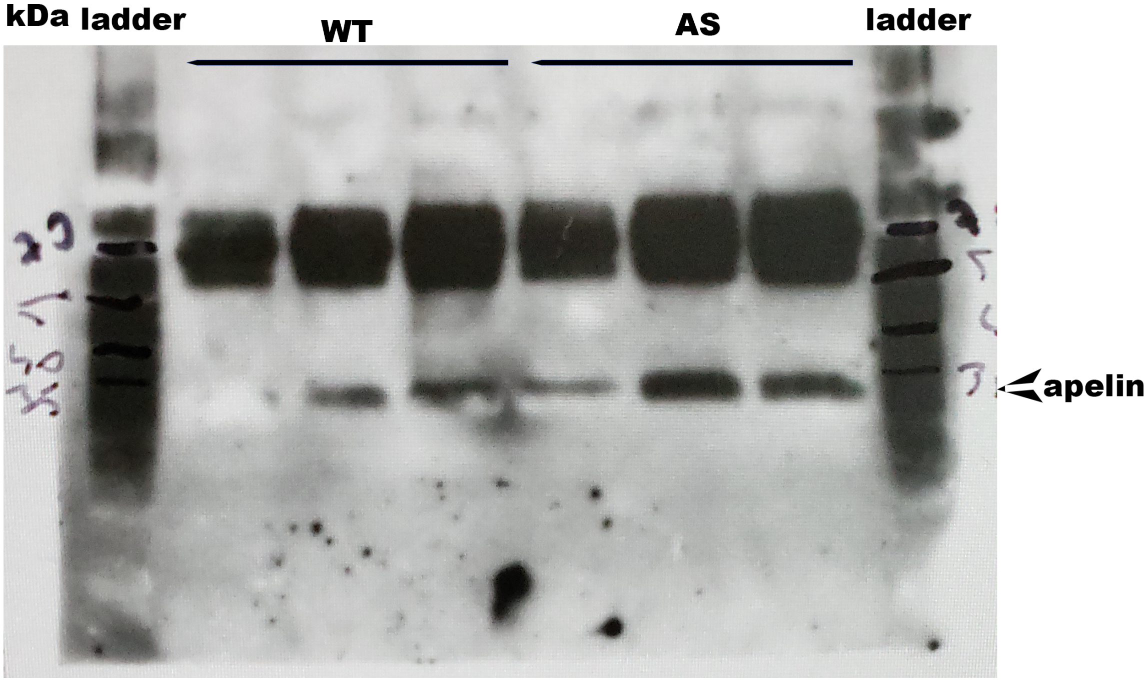

Fig. 6D: Western blot image showing protein expression for beta actin (of Apelin) in the WT and AS GEC

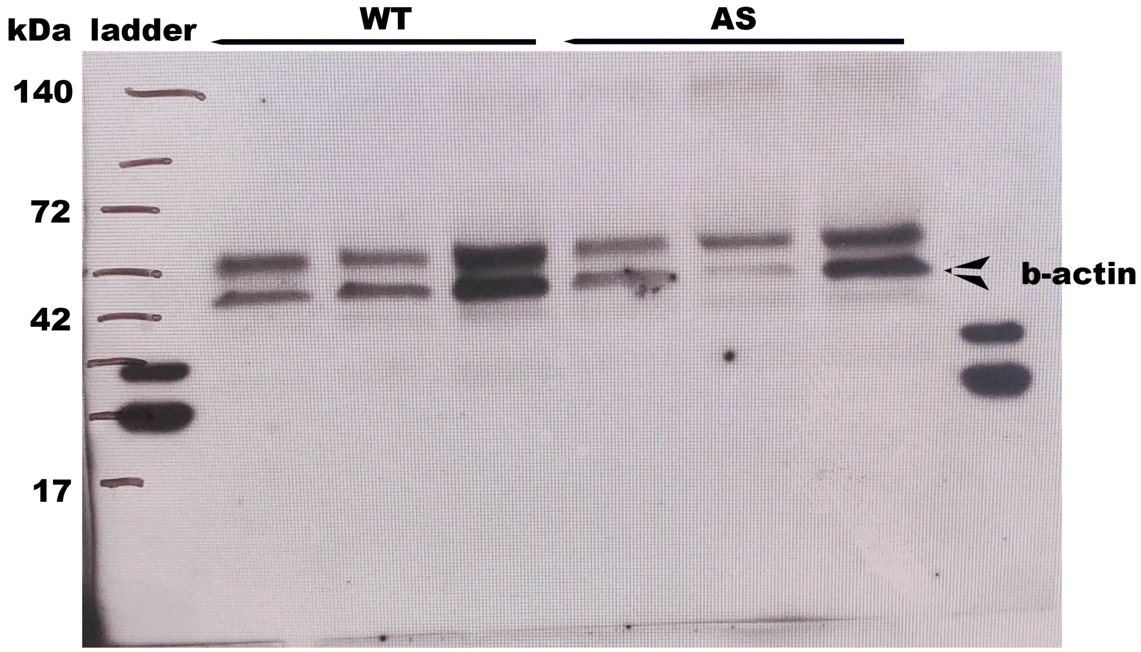

Fig. 6D: Western blot image showing protein expression for APJ in the WT and AS GEC

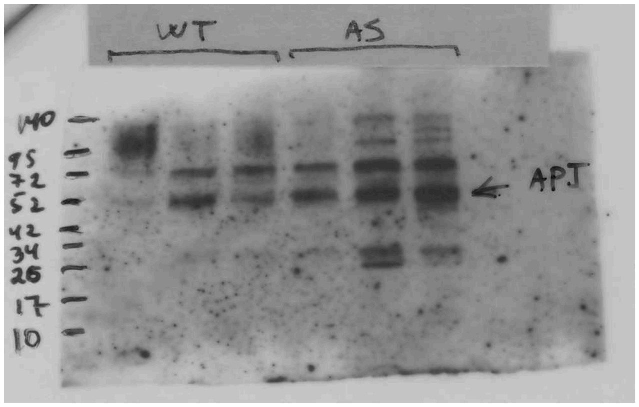

Fig. 6D: Western blot image showing protein expression for beta actin (of APJ) in WT and AS GEC

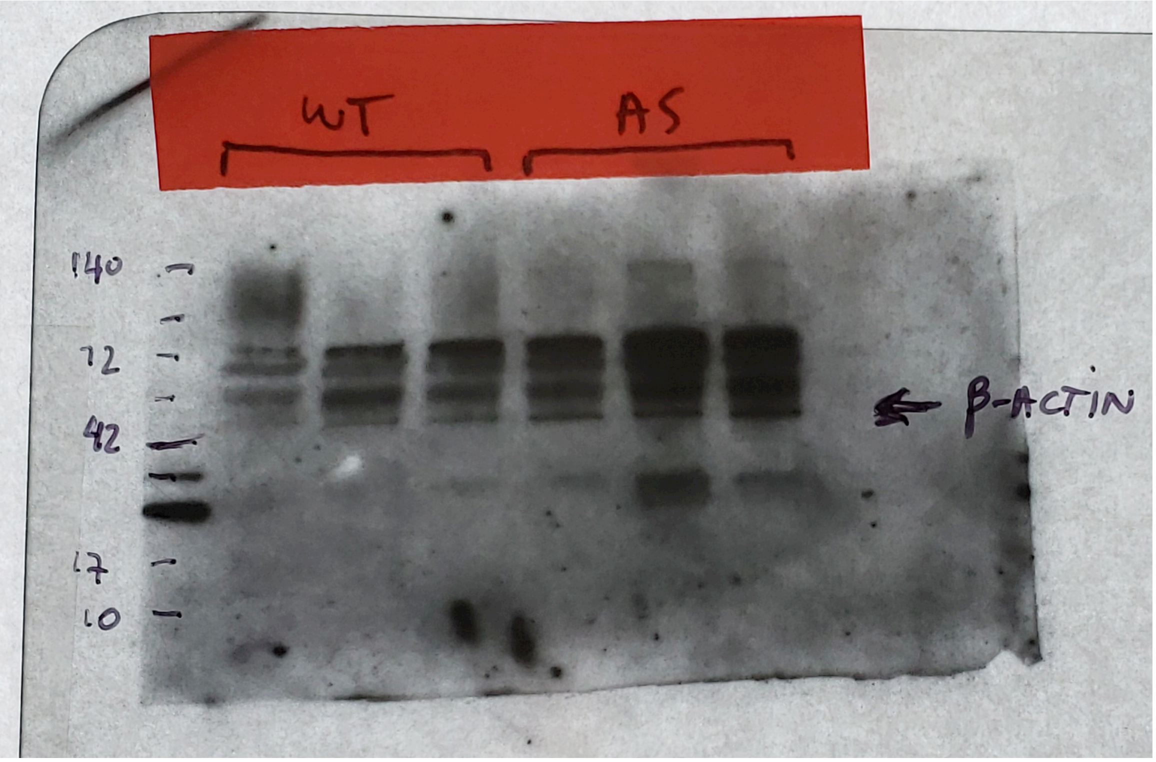

Fig. 6H: Western blot image showing protein expression for ICAM1 in human GEC untreated and GEC exposed to an apelin-13/pyr-apelin-13 dose

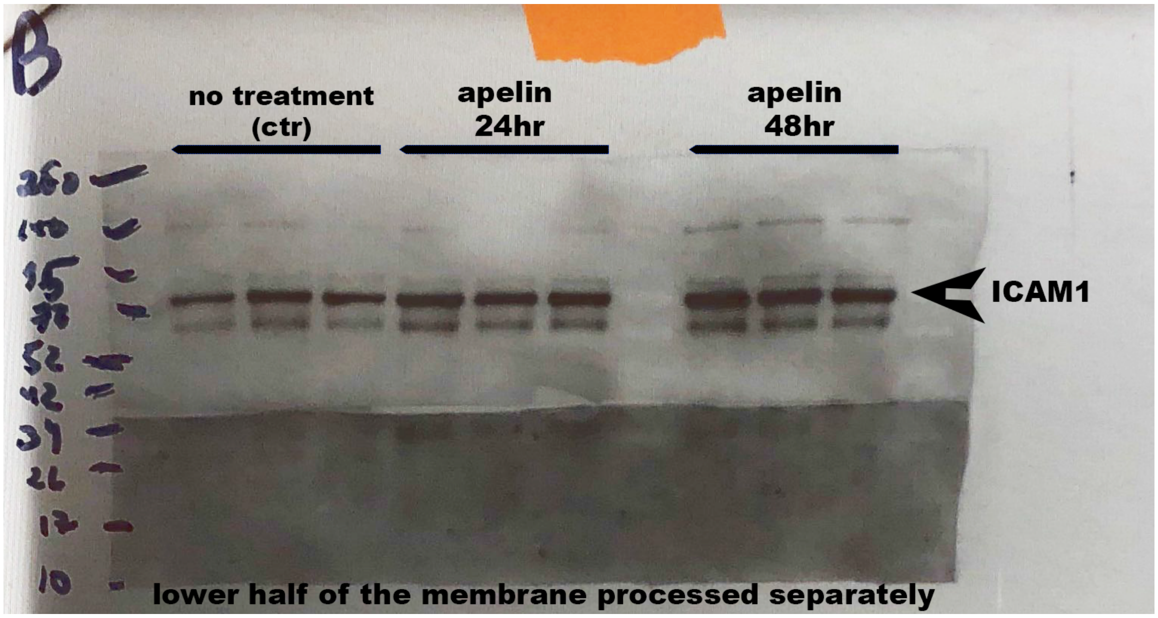

Fig. 6H: Western blot image showing protein expression for VCAM1 in human GEC untreated and GEC exposed to an apelin-13/pyr-apelin-13 dose

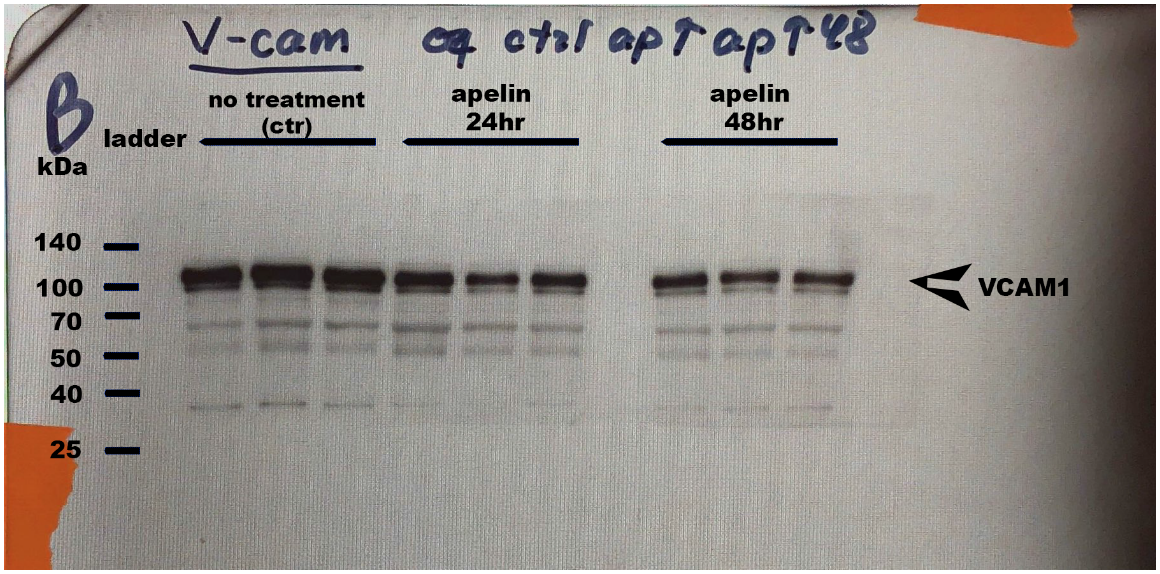

Fig. 6H: Western blot image showing protein expression for beta actin (of ICAM1 and VCAM1) in human GEC untreated and GEC exposed to an apelin-13/pyr-apelin-13 dose

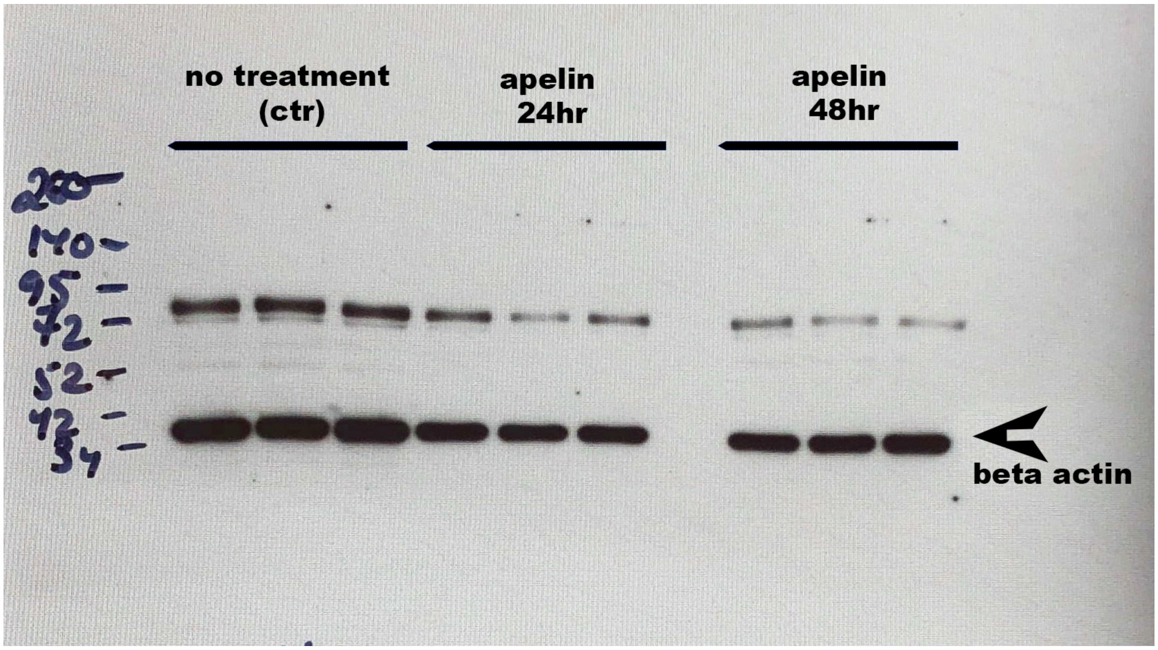

Suppl. Fig. 4: Western blot image showing protein expression for VCAM1 and beta actin in human GEC untreated and GEC exposed to an apelin-13 and pyr-apelin-13 dose independently

The last three lanes are unrelated to Suppl. Fig. 4.

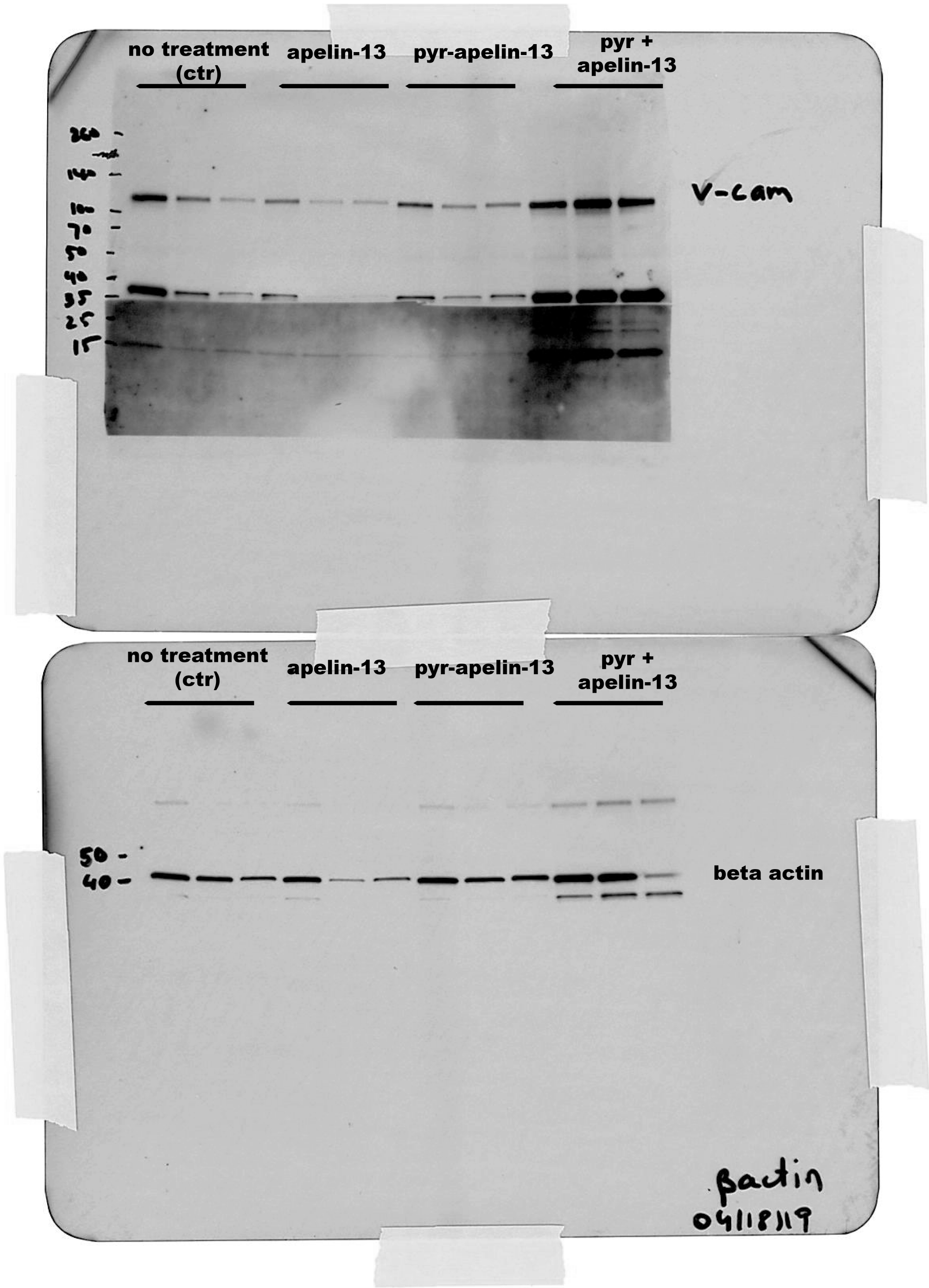

Suppl. Fig. 4: Western blot image showing protein expression for ICAM1 and beta actin in human GEC untreated and GEC exposed to an apelin-13 and pyr-apelin-13 dose independently

The last three lanes are unrelated to Suppl. Fig. 4.

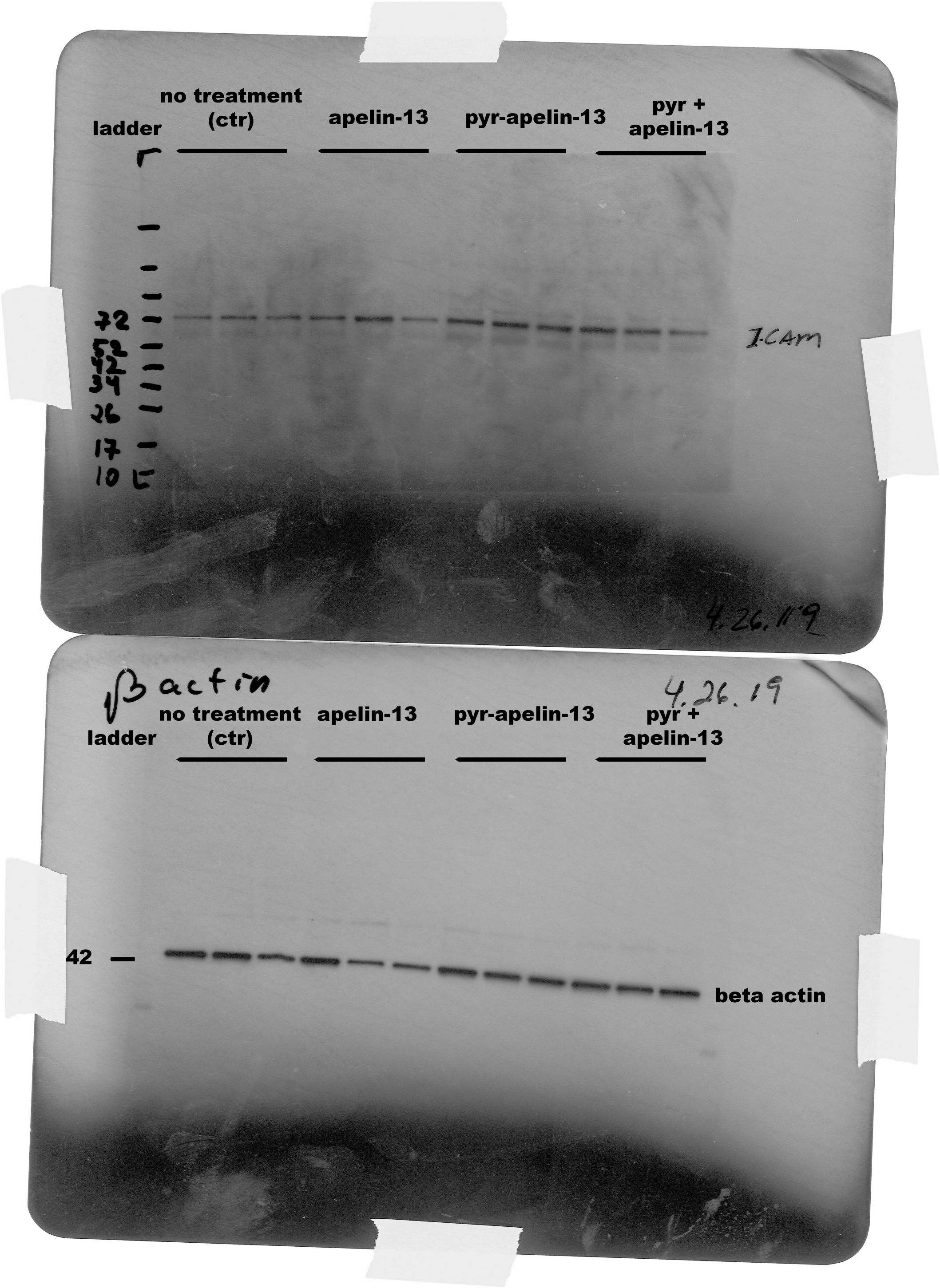

Supplement: Supplementary file 10 — Supplementary figures [file 41598_2020_67588_MOESM10_ESM.pdf]
